# Positive selection of efficient ethanol producers from xylose at 45 °C in the yeast *Ogataea polymorpha*

**DOI:** 10.1038/s41598-025-12204-2

**Published:** 2025-07-22

**Authors:** Roksolana Vasylyshyn, Justyna Ruchala, Kostyantyn Dmytruk, Andriy Sibirny

**Affiliations:** 1 Faculty of Biotechnology, Medical College, University of Rzeszow, 35-601 Rzeszów, Poland; 2 Department of Molecular Genetics and Biotechnology, Institute of Cell Biology, National Academy of Sciences of Ukraine, Lviv, 79005 Ukraine

**Keywords:** L-arabinose, Alcoholic fermentation, *API1* gene, *IRA1* gene, Reverse genetics, Metabolic engineering, Fungal biology, Fungal genetics, Gene expression

## Abstract

This research presents a method for the positive selection of mutants with improved xylose and L-arabinose fermentation in the thermotolerant, naturally xylose-utilizing yeast *Ogataea polymorpha* which is based on isolation of the mutants growing on L-arabinose as sole carbon and energy source. Whole-genome sequencing of the most efficient xylose-fermenting strain, A107, revealed mutations in the *API1* and *IRA1* genes, which are homologous to bacterial arabinose-5-phosphate isomerase and the Ras-GTPase activating domain in *Saccharomyces cerevisiae*, respectively. Disruption of the *IRA1* gene increased ethanol production during the fermentation of xylose and L-arabinose in *O. polymorpha* at 45 °C. Overexpression of the *API1* gene specifically enhanced L-arabinose fermentation without affecting xylose fermentation. The most productive mutant strain accumulated 20.91 g/L of ethanol in a xylose-containing medium at 45 °C, exceeding the ethanol accumulation level of the wild-type strain (0.40 g/L) by over 50 times. This strain holds potential for application in simultaneous saccharification and fermentation (SSF) processes.

**Supplementary Information:**

The online version contains supplementary material available at 10.1038/s41598-025-12204-2.

## Introduction

The production of biofuels from lignocellulosic renewable feedstocks continues to be a key objective in biotechnology^1,2^. One of unresolved challenges lies in developing the process of simultaneous saccharification and fermentation (SSF). This process integrates enzymatic hydrolysis of cellulose and hemicelluloses with the capture and conversion of liberated monosaccharides into ethanol, all within the same vessel^3,4^. As cellulases and hemicellulases optimally work at high temperatures (around 50 °C), microorganisms applied in the SSF process should preferably be thermotolerant and active at these elevated temperatures. Fermentation at high temperatures has several additional advantages, including reduced contamination risk, enhanced substrate solubility, increased fermentation rate and decreased cooling costs^5,6^.

Yeast organisms developed for lignocellulose processing are typically mesophilic, with optimal growth and fermentation temperatures around 30 °C (*Saccharomyces cerevisiae, Scheffersomyces stipitis, Spathaspora passalidarum*, etc.) and thus fail to meet the requirements for SSF^7–9^. Methylotrophic thermotolerant yeast *Ogataea polymorpha* maximally grows above 50 °C and could be suitable for use in the SSF process, especially since it naturally grows and ferments the second most abundant sugar of lignocellulose, xylose^6,10^. Wild-type strains of *O. polymorpha*, however, produce only negligible amounts of ethanol from this pentose.

The problem was partially resolved in our previous works, which resulted in strains with a 40-fold increase in ethanol production from xylose at 45 °C and no detectable accumulation of xylitol. This improvement was achieved through a combination of metabolic engineering (overexpression of the genes *XYL1*, *XYL2*, and *XYL3* of primary xylose metabolism; the genes *DAS1* and *TAL2*, coding for peroxisomal transketolase and transaldolase, respectively; knockout of the *CAT8* gene coding for a transcription activator; and engineering xylose reductase to reduce its affinity for NADPH) and random selection (isolation of mutants unable to grow in ethanol and those resistant to the anticancer drug 3-bromopyruvate)^11–14^. Further studies demonstrated the positive effects of overexpression of pyruvate decarboxylase (*PDC1*), cytosolic transketolase (*TKL1*), and transaldolase (*TAL1*), as well as transcription factors *MIG1, MIG2, HAP4-A, TUP1, AZF1*, and the hexose sensor *HXS1*, on xylose fermentation^14–16^. Furthermore, engineering the *O. polymorpha* hexose transporter Hxt1 relieved glucose repression of xylose utilization^17^.

In this work, we report for the first time the development of a simple positive selection method for *O. polymorpha* strains that accumulate 1.3 times more ethanol from xylose relative to the best available strain^14^. The method is based on positive selection for colonies growing on plates with L-arabinose as sole carbon and energy source. During xylose fermentation at 45 °C, the maximal ethanol titer reached 20.91 g/L, which is apparently the highest reported ethanol concentrations from xylose at this temperature. Our strains did not accumulate xylitol or other by-products, which represents a notable advantage compared to other engineered yeast strains^18^. The constructed here strain also accumulated visible amounts of ethanol from L-arabinose and elevated amounts of ethanol from lignocellulosic hydrolysates at 45 °C.

Whole-genome sequencing of the isolated most advanced mutant strain revealed mutations in the *API1* and *IRA1* genes, which encodes for arabinose-5-phosphate isomerase and a Ras-GTPase activating protein involved in the Ras-cAMP pathway, respectively. The *ira1Δ* mutant exhibited a 1.3-fold increase in ethanol production from xylose and a sixfold increase from L-arabinose compared to the parental strain, which was earlier engineered for advanced xylose fermentation. This highlights *IRA1* as a promising target for the metabolic engineering of improved ethanol producers from pentose sugars of lignocellulosic biomass. The *API1* gene, newly identified in *O. polymorpha* (and yeasts in general), was found to play a substantial role in L-arabinose assimilation and fermentation, as its overexpression substantially improved growth and elevated ethanol production from this pentose. These findings not only deepen our understanding of pentose metabolism in *O. polymorpha* but also suggest that the constructed strains are promising candidates for testing in simultaneous saccharification and fermentation (SSF) processes.

## Results

### Isolation of *O. polymorpha* strains with improved alcoholic fermentation of xylose and L-arabinose through a new positive selection method

**Fig. 1:**
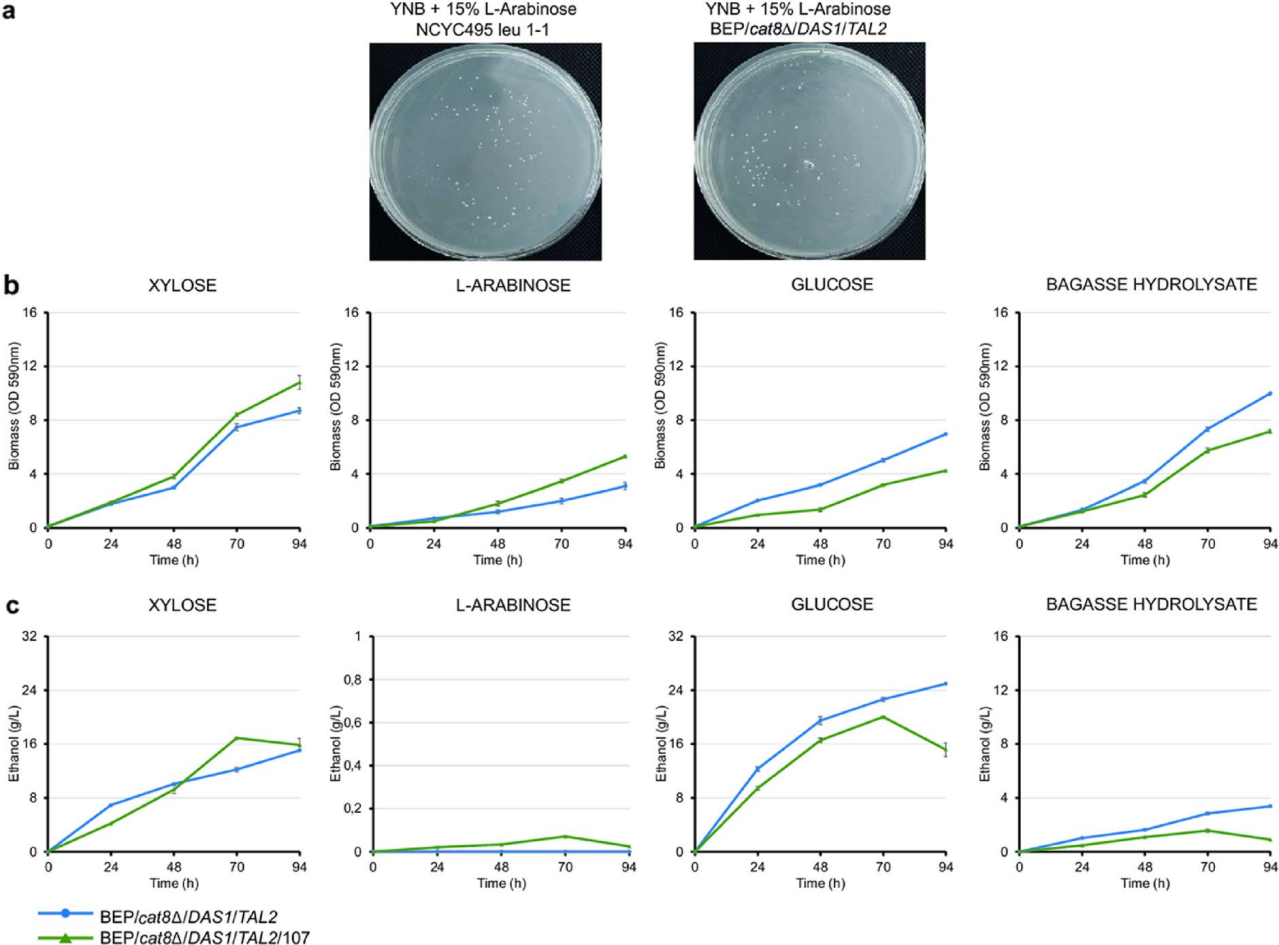
Growth, ethanol production, and selection of *O. polymorpha* mutants on different carbon sources. (**a**) Plating *O. polymorpha* UV-irradiated cells on a medium containing 15% L-arabinose. (**b**) Biomass accumulation of BEP/*cat8Δ/DAS1/TAL2* and BEP/*cat8Δ/DAS1/TAL2/107* mutant in YNB medium supplemented with 2% xylose, 2% L-arabinose, 2% glucose, or 50% bagasse hydrolysate during a growth test at 37 °C. (**c**) Ethanol production (g/L) by the parental *O. polymorpha* BEP/*cat8Δ/DAS1/TAL2* strain and one of the best UV-mutants BEP/*cat8Δ/DAS1/TAL2/107* during high-temperature alcoholic fermentation (45 °C) with 10% xylose, 10% L-arabinose, 10% glucose, or 50% bagasse hydrolysate. Error bars represent the standard error of the mean (SE), n = 3. In some cases, error bars are not visible due to the small magnitude of the error.

UV-mutagenized cells of *O. polymorpha* BEP/*cat8Δ/DAS1/TAL2* strain, an efficient ethanol producer from xylose^14^, and the wild-type strain NCYC495 leu1-1 were plated on minimal media containing 15% L-arabinose (Fig. 1a). This approach yielded, on average, 80 colonies capable of growing on L-arabinose plates. The growth kinetics of one of the selected strains, BEP/*cat8Δ/DAS1/TAL2/*107, are presented in Fig. 1b. The growth dynamics of the mutant were similar to those of the parental strain in xylose-containing media and were significantly improved (*P* = 0.024, one-way ANOVA, n = 3) in the medium with L-arabinose; however, its growth on glucose and lignocellulosic hydrolysate was reduced.

During high-temperature (45 °C) fermentation with 10% xylose as the sole carbon source, BEP/*cat8Δ/DAS1/TAL2/*107 mutant produced up to 16.9 g/L ethanol, compared to approximately 15.1 g/L from the parental strain (Fig. 1c). The selected mutant accumulated only trace amounts of ethanol during L-arabinose fermentation, reaching 0.07 g/L (Fig. 1c). In the glucose-containing minimal medium, a slight decrease in ethanol production by the mutant was observed compared to the parental BEP/*cat8Δ/DAS1/TAL2* strain (Fig. 1c). The mutant exhibited also a reduction in ethanol production (94 h) on hydrolysates, suggesting the increase in sensitivity of the obtained mutant to low pH levels in the medium (Fig. 1c). This hypothesis will be further explored in the following sections.

**Fig. 2:**
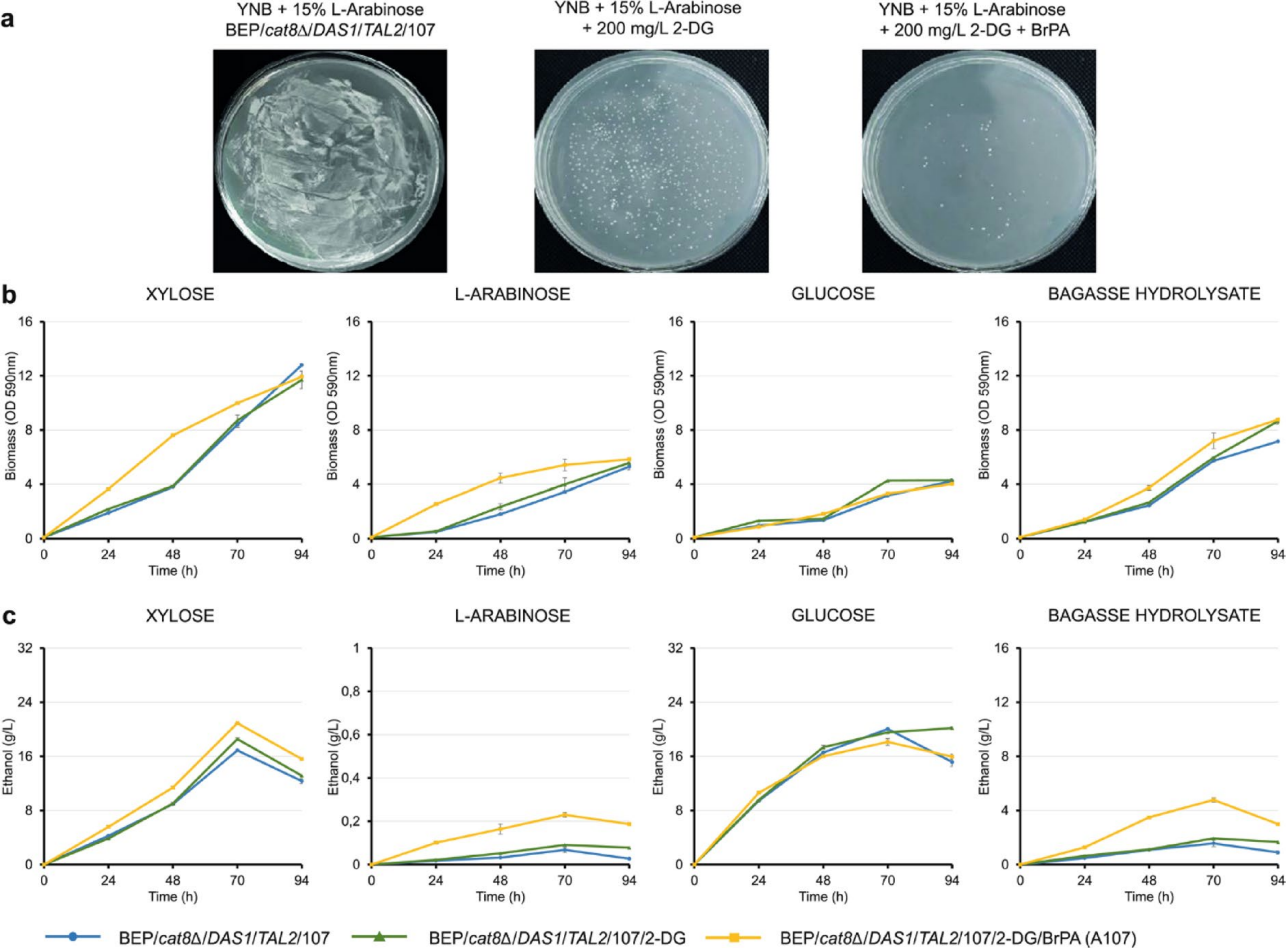
Growth, ethanol production, and selection of *O. polymorpha* mutants on different carbon sources. (**a**) Stepwise selection of BEP/*cat8Δ/DAS1/TAL2/*107 mutants resistant to 2-DG and BrPA on a medium containing 15% L-arabinose. (**b**) Biomass accumulation of BEP/*cat8Δ/DAS1/TAL2*/107, BEP/*cat8Δ/DAS1/TAL2/*107/2-DG and BEP/*cat8Δ/DAS1/TAL2/*107/2-DG/BrPA (A107) mutants in YNB medium supplemented with 2% xylose, 2% L-arabinose, 2% glucose, or 50% bagasse hydrolysate during a growth test at 37 °C. (**c**) Ethanol production (g/L) by the *O. polymorpha* selected mutants BEP/*cat8Δ/DAS1/TAL2*/107, BEP/*cat8Δ/DAS1/TAL2/*107/2-DG and BEP/*cat8Δ/DAS1/TAL2/*107/2-DG/BrPA (A107) during high-temperature alcoholic fermentation (45 °C) of 10% xylose, 10% L-arabinose, 10% glucose, or 50% bagasse hydrolysate. Error bars represent the standard error of the mean (SE), n = 3. In some cases, error bars are not visible due to the small magnitude of the error.

In the subsequent rounds of selection, the search for resistance to 2-deoxyglucose (2-DG) was employed. This deoxyglucose isomer has the hydroxyl group at the second position replaced by hydrogen. Resistance to this compound can result from mutations in structural or regulatory genes involved in glycolysis or sugar transport, potentially enhancing ethanol production^19,20^. After three days of incubation on 15% L-arabinose with 200 mg/L 2-DG, approximately 30 resistant BEP/*cat8Δ/DAS1/TAL2/*107/2-DG mutants were selected (Fig. 2a). Analysis of the 2-DG-resistant mutants identified one in which ethanol production during xylose fermentation at 45 °C was further increased to 18.6 g ethanol/L. However, L-arabinose fermentation remained minimal, reaching only about 0.1 g ethanol/L (Fig. 1e). An additional positive selection step for BEP/*cat8Δ/DAS1/TAL2/*107/2-DG strains was performed on medium with 0.05–0.11 mM 3-bromopyruvate (BrPA). BrPA inhibits multiple glycolytic enzymes, potentially affecting growth on glucose or xylose^21,22^. This selection approach was used by us with success for the isolation of *O. polymorpha* strains with improved xylose fermentation^13^. This time selection for BrPA resistance also proved to be highly effective, as we isolated the mutant BEP/*cat8Δ/DAS1/TAL2/*107/2-DG/BrPA (designated further as A107) accumulating 20–21 g of ethanol/L in 10% xylose medium (Fig. 2b and Table 2), which is 50 times higher than that of wild-type (0.4 g/L) and 1.67 times higher compared to the parental BEP/*cat8Δ/DAS1/TAL2* strain. To the best of our knowledge, this is the highest ethanol titer reported for yeast fermenting xylose at 45 °C, based on a comparison with previously published data^18^. Although mutants consumed xylose 1.3 times faster, based on the specific sugar consumption rate (SQR, g/L·OD·h) (72 h), a substantial portion of the sugar (approximately 29%) remained unutilized (Supplementary Fig. 1). Specific sugar consumption rates (SQR) were determined according to the “Methods” section.

The A107 mutant also showed increased ethanol production from L-arabinose, reaching 0.23 g/L. The parental strain had minimal capacity to metabolize L-arabinose, resulting in negligible ethanol production, whereas mutant A107 demonstrated improved L-arabinose utilization, consuming 5.5 g/L of the substrate within 94 h of alcoholic fermentation (Supplementary Fig. 1). Notably, the SQR (g/L·OD·h) increased threefold compared to the BEP/*cat8Δ/DAS1/TAL2* strain. The growth of the strain A107 in 50% lignocellulosic hydrolysate was similar to that of the parental strains BEP/*cat8Δ/DAS1/TAL2* whereas ethanol accumulation was substantially increased (from 2.84 g/L in strain BEP/*cat8Δ/DAS1/TAL2* to 4.78 g/L in strain A107) (Fig. 2b and c). Over 90% of xylose was consumed by the A107 mutant strain, whereas, despite the presence of arabinose in trace amounts (0.2 g/L) in the hydrolysate, no uptake was observed (Supplementary Fig. 1). Thus, A107 appeared to be a promising strain for studying the genetic and metabolic changes that enhance ethanol production from xylose in mutants capable of growing on L-arabinose.

### Identification, construction, and analysis of *O. polymorpha* yeast transformants with deletion and overexpression of the *API1* gene

First, we identified the changes that occurred in the genome of the selected A107 strain identified after its whole genome sequencing (Supplementary Table 1). The first mutation, which we consider to be relevant, is located on chromosome 2 at position 423410 and causes a nucleotide substitution from G to C in the gene (CDC) OGAPODRAFT_15612, resulting in an amino acid change from leucine (L) to phenylalanine (F) (Supplementary Fig. 2). The probable product of this gene is arabinose-5-phosphate isomerase. This enzyme catalyzes the reversible isomerization of ribulose-5-phosphate (Ru5P) to arabinose-5-phosphate (Ar5P) for the production of 3-deoxy-D-manno-octulosonic acid 8-phosphate (KDO), a component of bacterial lipopolysaccharide (LPS) in Gram-negative bacteria^23^. While the function of APIs is inherently defined, the role they serve within a bacterium can vary. Moreover, the essentiality of individual Api1 proteins is unclear^24^. There is no direct link between arabinose-5-phosphate isomerase (*API1*) and L-arabinose metabolism. In yeasts, the gene coding for arabinose-5-phosphate isomerase was not annotated. Although this mutation was considered potentially relevant, the expression level of *API1* remained unchanged in the evolved A107 strain (Supplementary Fig. 3). Therefore, instead of testing the effect of the specific mutation, we aimed to determine whether *API1* itself plays a functional role in L-arabinose metabolism. To this end, we employed a reverse genetics approach by constructing plasmids containing expression modules for the homologous *API1* gene from *O. polymorpha*, as well as a deletion construct to generate knockout mutants.

**Fig. 3:**
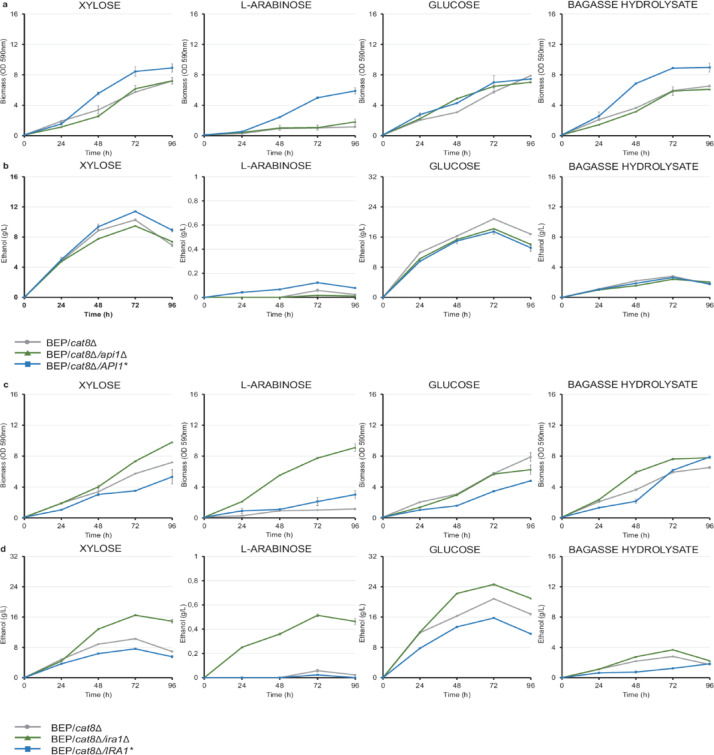
Biomass accumulation by BEP/*cat8Δ*, BEP/*cat8∆/api1∆* and BEP/cat8∆/API1*, BEP/*cat8Δ/ira1Δ* and BEP/cat8Δ/IRA1* and ethanol production by these strains. (**a**) Biomass accumulation by BEP/*cat8Δ*, BEP/*cat8∆/api1∆* and BEP/cat8Δ/API1* in minimal medium supplemented with 2% xylose, 2% L-arabinose, 2% glucose, and 50% bagasse hydrolysate at 37 °C and (**b**) ethanol production by these strains during the alcoholic fermentation of 10% xylose, 10% L-arabinose, 10% glucose, and 50% bagasse hydrolysate at 45 °C. (**c**) Biomass accumulation by BEP/*cat8Δ*, BEP/*cat8∆/ira1∆,* BEP/cat8∆/IRA1* and A107 in minimal medium supplemented with 2% xylose, 2% L-arabinose, 2% glucose, and 50% bagasse hydrolysate at 37 °C and (**d**) ethanol production by these strains during the alcoholic fermentation of 10% xylose, 10% L-arabinose, 10% glucose, and 50% bagasse hydrolysate at 45 °C. Error bars represent the standard error of the mean (SE), n = 3. In some cases, error bars are not visible due to the small magnitude of the error.

Deletion and overexpression of the *API1* gene in *O. polymorpha* were performed in the background of the BEP/*cat8Δ* strain, an improved ethanol producer from xylose^12^. The constructed strains BEP/*cat8Δ/api1Δ* and BEP/cat8Δ/API1* were analyzed for *API1* gene expression, as well as their growth, fermentation, and consumption of glucose, xylose, arabinose, and hydrolysates under various conditions. The reduced expression of the *API1* gene in BEP/*cat8Δ/api1Δ* confirmed the deletion of the gene (*P* = 0.017, unpaired two-tailed t-test, n = 3), (Table 1). It was found that *API1* gene deletion did not affect the biomass accumulation of the recombinant strain on different carbon sources (Fig. 3a). This also had a minimal inhibitory impact on ethanol fermentation of the selected sugars, particularly of glucose (Fig. 3b). Sugar concentration (g/L) profiles in the BEP/*cat8Δ/api1Δ* strain were similar to those observed in the parental BEP/cat8Δ strain (Supplementary Fig. 1).

**Table 1:** (a) Relative expression levels of *API1* and *IRA1* genes expression in BEP/*cat8∆/api1∆* and BEP/cat8∆/API1*, BEP/cat8∆/ira1∆ and BEP/cat8∆/IRA1* strains versus the recipient strain BEP/*cat8Δ* at the third day of 10% xylose fermentation at 45 °C. (b) Relative expression levels of the inducible *IRA1* gene under control the *YNR1* promoter in BEP/cat8Δ/IRA1* strain compared to parental strain BEP/cat8Δ during cultivation in media with 10% xylose and ammonium sulfate or nitrate, and during sequential transfer from one medium to another. Values represent mean ± 95% confidence interval (CI) from three biological replicates. Details of CI calculation are provided in the “Methods” Section.

Yeast strains	*∆∆*Ct	
	Genes	
	*API1*	*IRA1*
a		
BEP/*cat8∆/api1∆*/BEP/*cat8∆*	0.54 ± 0.258	-
BEP/cat8∆/API1*/BEP/*cat8∆*	4.75 ± 0.199	-
BEP/*cat8∆/ira1∆/*BEP/*cat8∆*	-	0.62 ± 0.090
BEP/cat8∆/IRA1*/BEP/*cat8∆*	-	14.5 ± 0.902

Strain with overexpression of *IRA1* gene under control of *YNR1* promoter	*∆∆*Ct	
	Medium	
	*NO*_*3*_^*−*^	*NH*_*4*_^+^
b		
BEP/cat8∆/IRA1*/BEP/*cat8∆*	9.46 ± 0.345	0.93 ± 0.197
BEP/cat8∆/IRA1*_NO_3_^−^/BEP/*cat8∆*	12.8 ± 0.657	0.85 ± 0.262
BEP/cat8∆/IRA1*_NH_4_^+^/BEP/*cat8∆*	13.2 ± 0.248	0.7 ± 0.043

We also constructed a strain overexpressing the *API1* gene under the control of the strong constitutive *GAP* promoter from the gene encoding glyceraldehyde-3-phosphate dehydrogenase. Overexpression was confirmed by qRT-PCR. The BEP/cat8Δ/API1* strain showed a 4.8-fold increase in *API1* expression (*P* = 0.0002, unpaired two-tailed t-test, n = 3), (Table 1). The BEP/cat8Δ/API1* strain exhibited 1.5-, 4.9-, and 1.2-fold higher biomass accumulation (72 h) on 2% xylose, 2% L-arabinose, and 2% glucose, respectively, compared to BEP/*cat8Δ*. Additionally, strains with *API1* overexpression demonstrated a 1.5-fold increase in growth after 72 h on 50% bagasse hydrolysates (Fig. 3a). The BEP/cat8Δ/API1* strain possessed 1.1- and twofold increased ethanol production (72 h) on xylose and L-arabinose, reaching 11.4- and 0.12 g ethanol/L, respectively (Table 2). Additionally, overexpression negatively affects ethanol production on glucose and hydrolysates. (Fig. 3b). Xylose utilization was similar to that of the parental strain (Supplementary Fig. 1), whereas the amount of arabinose consumed (g/L) and the SQR (g/L·OD·h) increased twofold. The observed results clearly show the role of the *API1* gene in the yeast *O. polymorpha* involved specifically in L-arabinose metabolism and fermentation*.*

**Table 2:** Main parameters of xylose fermentation at 45 °C by the tested A107 mutant strain and yeast transformants *O. polymorpha* with *API1* and *IRA1* genes deletion and overexpression.

Strain	Ethanol (g/L)	Ethanol yield (g/g consumed xylose)	Theoretical yield (%)	Ethanol specific production rate (g/g biomass/h)	Ethanol productivity (g/L/h)
a					
A107	20.91 ± 0.138	0.35 ± 0.009	68.6 ± 1.77	0.126 ± 0.001	0.290 ± 0.002
BEP/*cat8Δ*	10.28 ± 0.141	0.25 ± 0.007	49.0 ± 1.14	0.071 ± 0.001	0.150 ± 0.002
BEP/*cat8∆/api1∆*	9.47 ± 0.065	0.28 ± 0.007	54.9 ± 1.42	0.063 ± 0.001	0.131 ± 0.001
BEP/cat8∆/API1*	11.40 ± 0.076	0.25 ± 0.007	49.0 ± 1.27	0.075 ± 0.001	0.158 ± 0.001
BEP/*cat8∆/ira1∆*	16.50 ± 0.173	0.33 ± 0.009	64.7 ± 1.76	0.101 ± 0.001	0.229 ± 0,003
BEP/cat8∆/IRA1*	7.61 ± 0.067	0.23 ± 0.006	45.1 ± 1.18	0.050 ± 0.004	0.106 ± 0.001
b					
A107	0.251 ± 0.046	0.055 ± 0.013	10.8 ± 2.5	0.002 ± 0.001	0.004 ± 0.001
BEP/*cat8Δ*	0.055 ± 0.043	0.008 ± 0.002	1.57 ± 1.2	0.001 ± 0.001	0.001 ± 0.001
BEP/*cat8∆/api1∆*	0.013 ± 0.008	0.002 ± 0.001	0.39 ± 0.27	0.000 ± 0.001	0.000 ± 0.000
BEP/cat8∆/API1*	0.132 ± 0.031	0.010 ± 0.007	1.96 ± 0.46	0.001 ± 0.001	0.002 ± 0.001
BEP/*cat8∆/ira1∆*	0.520 ± 0.052	0.043 ± 0.021	8.41 ± 0.84	0.003 ± 0.002	0.007 ± 0.001
BEP/cat8∆/IRA1*	0.025 ± 0.005	0.007 ± 0.004	1.37 ± 0.27	0.000 ± 0.001	0.000 ± 0.000

Data of ethanol yield and ethanol (g/L) are represented on YNB medium supplemented with (a) 10% of xylose or (b) 10% L-arabinose on 72 h of fermentation. Values represent mean ± 95% confidence interval (CI) from three biological replicates. Details of CI calculation are provided in the “Methods” Section.

### Identification, construction, and analysis of *O. polymorpha* yeast transformants with deletion and overexpression of the *IRA1* gene

The second mutation occurs on chromosome 3 at positions 958485–958486, involving an adenine insertion (reverse) in the gene (CDC) OGAPODRAFT_93633, leading to a frameshift and the introduction of stop codons (Supplementary Fig. 2). The likely product of this gene is an activating Ras-GTPase protein domain homologous to RasGAP proteins Ira1 and Ira2 in *S. cerevisiae*, which are negative regulators of the Ras-cAMP signaling pathway. The frameshift resulted in a nonsense mutation and the loss of 546 amino acids from the C-terminal fragment of the Ira1 polypeptide chain in *O. polymorpha*. A similar mutation in the *IRA2* gene of *S. cerevisiae* removes 152 C-terminal amino acids, a region previously identified as critical for Ira2 stability^25^.

Expression of *O. polymorpha IRA1* gene was analyzed to investigate the genetic and metabolic changes that led to increased ethanol production from xylose and L-arabinose in the mutants capable of growing well on L-arabinose. The expression of the *IRA1* gene was reduced in BEP/*cat8Δ/ira1Δ*, confirming deletion of the gene (*P* = 0.003, unpaired two-tailed t-test, n = 3) (Table 1). Deletion of the *IRA1* gene resulted in a 1.3-, 7.6-, and 1.3-fold increase in biomass accumulation rate (72 h) on 2% xylose, 2% L-arabinose, and 50% bagasse hydrolysate, respectively (Fig. 3c). After 96 h of cultivation on 2% glucose, the biomass accumulation in the BEP/*cat8Δ/ira1Δ* strain was not substantially different from that of the parental BEP/*cat8Δ* strain, with the observed variation falling within the limits of experimental error (Fig. 3c). A total of 16.5 g/L of ethanol was accumulated during BEP/*cat8Δ/ira1Δ* high-temperature ethanol fermentation with 10% xylose (45 °C), which is 1.6 times higher than that of the parental strain (Fig. 3d and Table 2). We found that ethanol production in the BEP/*cat8Δ/ira1Δ* strains in medium with 10% L-arabinose reached 0.5 g/L, the highest value reported to date for this yeast species. The deletion also impacted ethanol production from glucose and hydrolysates, increasing production by 1.2- and 1.3-fold, respectively (Fig. 3d). Additionally, the obtained strains consumed 1.5 times more xylose than the parental strain. However, 38 g/L of xylose remained in the medium by the 96th hour of fermentation (Supplementary Fig. 1). L-arabinose utilization was minimal, with only 12.6 g/L consumed by the end of fermentation. However, the L-arabinose consumption rate (SQR, g/L·OD·h), calculated based on sugar utilization and biomass accumulation over time, was doubled compared to the control. In the bagasse hydrolysate medium, the *IRA1* deletion strain exhibited complete glucose consumption, similar to the parental BEP/*cat8Δ* strain. The deletion strains utilized over 90% of the available xylose, however, despite the presence of trace amounts of L-arabinose (0.2 g/L) in the hydrolysate, no detectable uptake was observed (Supplementary Fig. 1).

Repeated attempts to generate mutants overexpressing the *IRA1* gene under the control of a strong constitutive *GAP* promoter at the beginning were unsuccessful. We hypothesized that such *O. polymorpha* mutants could be lethal. Consequently, we decided to create a conditional mutant that expresses the *IRA1* gene under the control of a strongly inducible promoter by replacing the *GAP* promoter with the promoter of the *O. polymorpha YNR1* gene, which is repressed by ammonium and strongly induced by nitrate^26^. A similar approach earlier was successfully applied in our previous work^14^. The resulting transformants were grown in mineral medium (with ammonium sulfate) and glucose or xylose as the carbon source. However, these strains exhibited limited growth on nitrate medium (Supplementary Fig. 3).

We subsequently confirmed the inducible regulation of the *IRA1* gene under the control of the *YNR1* promoter through qRT-PCR (*P* = 0.0001, unpaired two-tailed t-test, n = 3). Moreover, we demonstrated that strains pre-cultivated on ammonium sulfate and then transferred to nitrate exhibited 13.2-fold increased expression of the *IRA1* gene (*P* = 2.36 × 10^− 5^, unpaired two-tailed t-test, n = 3) (Table 1). Conversely, expression decreased when cultures were transferred from nitrate to ammonium sulfate, while transferring from nitrate to nitrate further increased expression by 1.4-fold (*P* = 0.0002, unpaired two-tailed t-test, n = 3) (Table 1). When transferred from ammonium sulfate to ammonium sulfate, the gene expression remained switched off, with background levels of expression possibly related to leakage from this type of promoter^27^. Furthermore, we continued our efforts to obtain recombinant BEP/cat8Δ/IRA1* strains expressing *IRA1* under the *GAP* promoter. Ultimately, we succeeded in obtaining 7 strains with severely impaired growth regardless of the carbon source used. After the stabilization process, we lost two of seven strains. In the remaining stable transformants, we measured the *IRA1* expression level, which was found to be 14.5 times higher than in the parental BEP/*cat8Δ* strain (*P* = 0.0002, unpaired two-tailed t-test, n = 3) (Table 1). The ethanol production in xylose and ammonium sulfate media for recombinant BEP/cat8Δ/IRA1* strains with the *IRA1* gene under the *YNR1* promoter did not differ from that of the parental BEP/cat8Δ strain, apparently due to the repression of *IRA1* by ammonium sulfate (Supplementary Fig. 4). Strains with the *IRA1* gene under the GAP promoter showed reduced biomass accumulation during the growth assay at 37 °C in media containing 2% xylose or 2% glucose. In both media, BEP/*cat8Δ* consistently outperformed BEP/*cat8∆/IRA1* in terms of growth, exhibiting slightly higher specific growth rates during the first 48 h (μ = 0.0736 h^−1^ vs. 0.0712 h^−1^ in xylose; μ = 0.0716 h^−1^ vs. 0.0574 h^−1^ in glucose) and greater overall biomass accumulation (OD_590_ = 7.19 vs. 5.32 in xylose; OD_590_ = 7.89 vs. 4.80 in glucose), indicating superior growth performance of the parental strain (Fig. 2c). In a medium with hydrolysates, the improved growth of BEP/cat8Δ/IRA1* strains can be attributed to enhanced stress resistance due to reduced PKA activity, potentially improving the cells ability to withstand stress factors during high-temperature fermentation of lignocellulosic hydrolysates. Slower growth during the 48-h growth test (μ = 0.077 h^−1^ for BEP/*cat8∆* vs. μ = 0.062 h^−1^ for BEP/cat8∆/IRA1*) may have also allowed for a more balanced energy distribution, enhancing survival under nutrient-limited or stressful conditions. Our hypotheses will be confirmed in the following section. At the same time, these strains exhibited 1.4-, 1.3-, or 2.3-fold reduced ethanol production (72 h) from 10% xylose, 10% glucose, or 50% bagasse hydrolysates and were unable to ferment 10% L-arabinose (Fig. 2d) during high-temperature alcoholic fermentation at 45 °C. Thus, our findings indicate that *IRA1* overexpression in *O. polymorpha* compromises growth performance and contributes to strain degeneration during prolonged cultivation. Similar conclusions have been drawn in other studies that investigated growth regulation through the inhibition or activation of the Ras-cAMP signaling system^28,29^.

## Functional analysis of genetic changes in the *O. polymorpha* adaptation to high xylose concentrations and environmental stress conditions

**Fig. 4:**
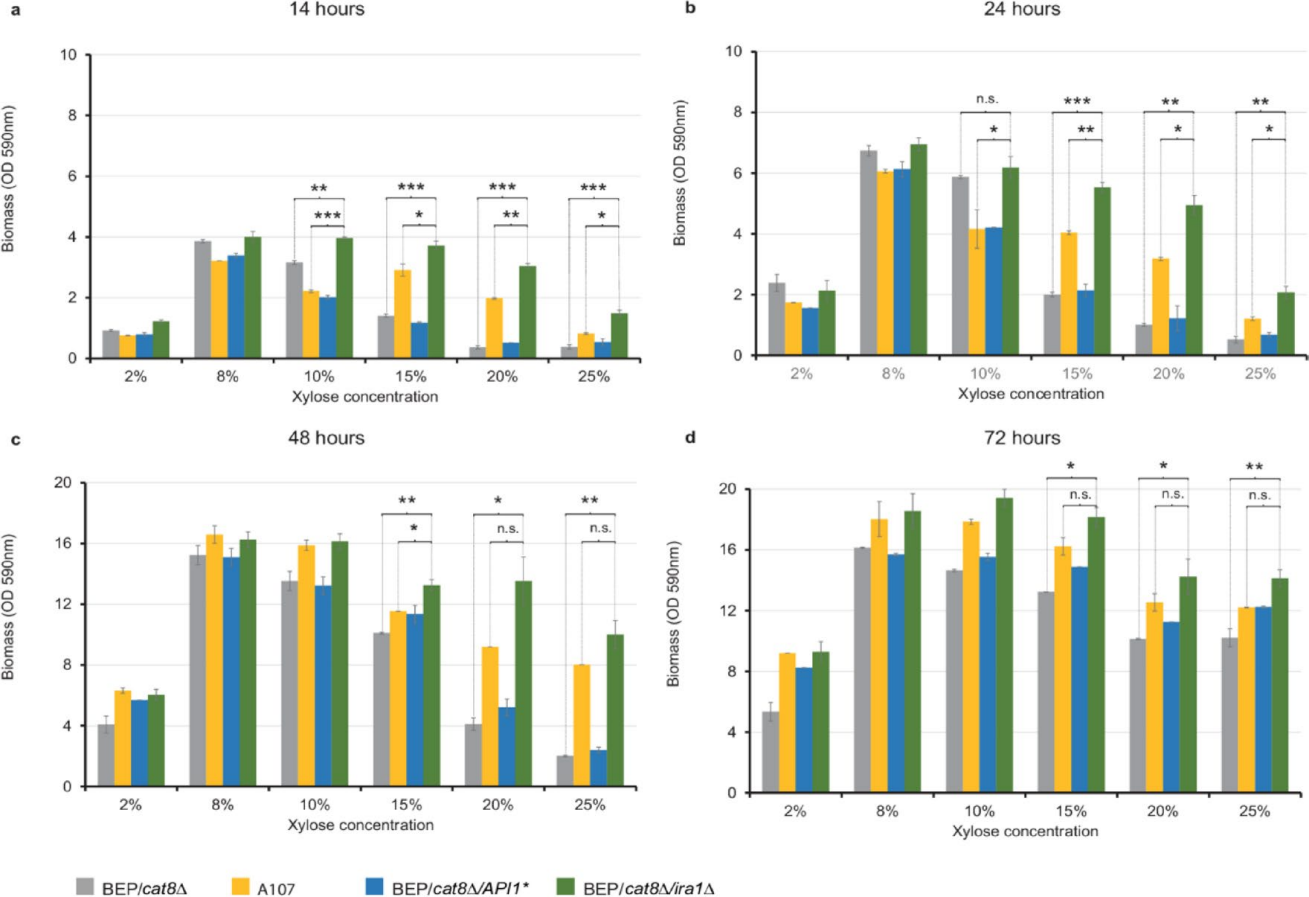
Growth kinetics in conditions of elevated xylose concentrations. Growth kinetics of parental BEP/*cat8Δ* strain, A107 mutant strain and yeast transformants *O. polymorpha* BEP/cat8∆/API1* and BEP/*cat8∆/ira1∆* in conditions of elevated xylose concentrations, performed at 37 °C for (a) 14 h; (b) 24 h; (c) 48 h; (d) 72 h. Error bars represent the standard error of the mean (SE). In some cases, error bars are not visible due to the small magnitude of the error. Statistical significance is indicated by asterisks: * *P* ≤ 0.05, ** *P* ≤ 0.01, *** *P* ≤ 0.001, calculated using unpaired two-tailed *t*-tests (*n* = 3). Dashed brackets denote the specific pairs of strains that were statistically compared.

At xylose concentrations of 8% and below, the specific growth rates of the BEP/*cat8∆*, A107, BEP/cat8∆/API1*, and BEP/*cat8∆/ira1∆* strains were relatively similar, with specific growth rates ranging from 0.0304 h^−1^ to 0.0430 h^−1^ or 0.0247 h^−1^ to 0.0297 h^−1^ (14–72 h), and the overall biomass accumulation positively correlated with sugar availability (Fig. 4 and Supplementary Table 2). However, when the xylose concentration exceeded 8%, biomass accumulation deteriorated rapidly during the first 48 h of the experiment in the BEP/*cat8∆*, A107, and BEP/cat8∆/API1* strains (Fig. 4), despite stable or even increased specific growth rates (Supplementary Table 2). In contrast, the BEP/*cat8∆/ira1∆* strain exhibited a less pronounced decrease in biomass accumulation with increasing xylose concentrations, with growth rates remaining stable throughout the growth test (μ = 0.265 h^−1^ to 0.368 h^−1^, 14–72 h), and even increasing (0.0419 h^−1^ to 0.0657 h^−1^, 24–48 h) at 20% and 25% xylose (Supplementary Table 2), indicating enhanced tolerance to sugar-induced stress. These data suggest that growth inhibition at high concentrations is specific to xylose and likely results from constitutive activation of metabolic pathways in strains lacking *IRA1*, which may impair their normal response to nutritional conditions.

**Fig. 5:**
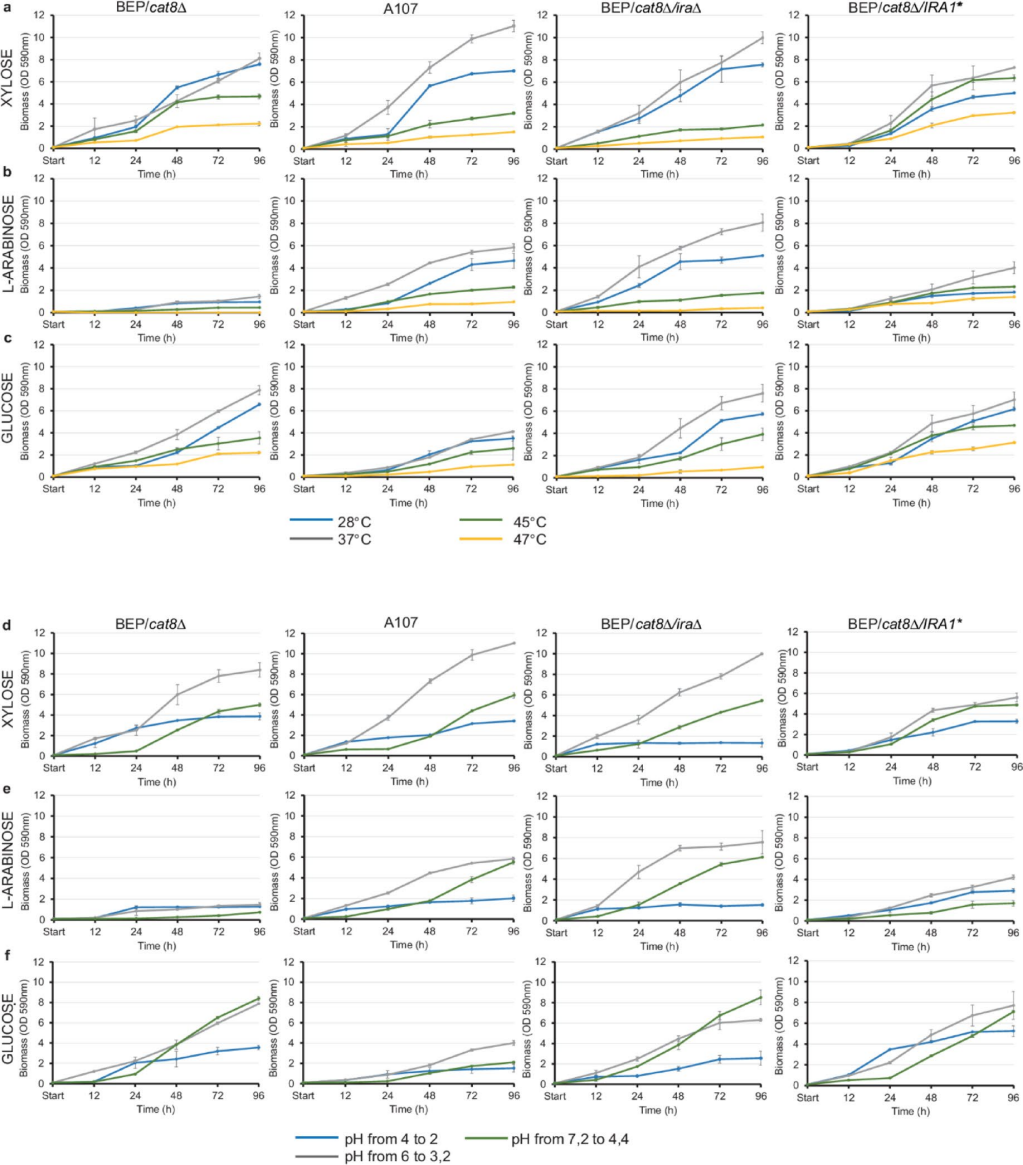
Biomass accumulation of four *O. polymorpha* strains at different temperatures and pH conditions. (**a**) Biomass accumulation of three *O. polymorpha* strains (BEP/*cat8Δ*, A107, BEP/cat8Δ/IRA1*, and BEP/*cat8Δ/ira1Δ*) in media containing 2% xylose, (**b**) 2% L-arabinose, (**c**) and 2% glucose at different temperatures (28 °C, 37 °C, 45 °C, 47 °C). Biomass accumulation of four *O. polymorpha* strains (BEP/*cat8Δ*, A107, BEP/cat8Δ/IRA1*, and BEP/*cat8Δ/ira1Δ*) in media containing (**d**) 2% xylose, (**e**) 2% L-arabinose and (**f**) 2% glucose at different pH conditions (4, 6, or 7.2). For medium with initial pH 4, the pH dropped to 2, for pH 6 it dropped to 3.2, and for pH 7.2, the pH reached 4.4 by the 96th hour of the experiment. Error bars represent the standard error of the mean (SE). In some cases, error bars are not visible due to the small magnitude of the error.

Deletion of one or both *IRA* genes makes *S. cerevisiae* cells sensitive to heat shock, low pH, and nitrogen starvation due to impaired G1 arrest under nutrient depletion^25^. Based on these findings, we decided to study the effects of temperature and pH on the growth rates of recombinant strains with *IRA1* deletion and overexpression. The strains display similar growth patterns on xylose. However, A107 and BEP/*cat8Δ/ira1Δ* revealed the highest biomass accumulation at 28 °C and 37 °C (OD_590_ 11 and OD_590_ 10, respectively, after 96 h of growth), while their growth sharply declines at 45 °C and 47 °C (OD_590_ 1.5 and OD_590_ 1.1, respectively, after 96 h of growth) (Fig. 5a).

Overexpression of the *IRA1* gene in the BEP/cat8Δ/IRA1* strain enabled growth on xylose even at 45 °C and 47 °C (OD_590_ 6.2 and OD_590_ 3 after 72 h of growth), highlighting the potential role of *IRA1* in stress tolerance, particularly under high-temperature conditions. In the L-arabinose medium, A107 and BEP/*cat8Δ/ira1Δ* strains accumulated substantial biomass, reaching OD_590_ values of 5.4 and 7.2, respectively, after 72 h of cultivation at 37 °C. During the exponential growth phase (24–48 h), their specific growth rates were 0.0235 h^−1^ for A107 and 0.0143 h^−1^ for BEP/*cat8Δ/ira1Δ*, further supporting their ability to grow efficiently on L-arabinose as the sole carbon source under the tested conditions (Fig. 5b). A107 has been identified as a mutant with activated L-arabinose fermentation, so these results are expected. Biomass accumulation on glucose was not notably different among the BEP/*cat8Δ*, BEP/*cat8Δ/ira1Δ*, and BEP/*cat8Δ/IRA1* strains at 28 °C, 37 °C, and even 45 °C, indicating a high level of adaptation to glucose metabolism (Fig. 5c). However, at 47 °C, the BEP/*cat8Δ/ira1Δ* strain exhibited the lowest biomass accumulation (OD_590_ = 0.7 after 72 h of growth), likely due to increased thermal sensitivity resulting from impaired cellular stress response pathways. Strain A107 exhibited the lowest biomass accumulation compared to the other strains across all tested sugars, which may be attributed to a heightened sensitivity of the mutant to glucose.

Acid pretreatment is a widely used method for processing lignocellulosic biomass^30^. The hydrolysates used by us typically have a pH range between 4.5 and 5.5. Three initial pH values (4, 6, and 7.2) were used in the experiment. The acidic environment (pH 4) negatively affects all strains, with a particularly strong impact on growth in xylose and arabinose. This can be attributed to increased cellular sensitivity to stress, which reduces metabolic activity and viability. The strain with the *IRA1* deletion (BEP/*cat8Δ/ira1Δ*) is the most sensitive to low pH (OD_590_ 1.3 on xylose and OD_590_ 1.5 on L-arabinose at 37 °C for 96 h) (Fig. 5d and e), which is a critical factor when working with hydrolysates. We suggest that the absence of *IRA1* leads to an imbalance in the cAMP-PKA signaling pathway, resulting in increased cell sensitivity. Overexpression of *IRA1* (BEP/cat8Δ/IRA1*) yielded one of the highest biomass accumulation under elevated stress conditions in 2% xylose, 2% L-arabinose, and 2% glucose (OD_590_ 3.3, OD_590_ 2.9, and OD_590_ 5.2, respectively, at an initial pH of 4 after 96 h of growth), confirming the importance of this gene in adaptation to high acidity or temperature stresses in *O. polymorpha*.

## Discussion

This study aimed to construct and characterize recombinant strains of the thermotolerant yeast *O. polymorpha* with enhanced ability to ferment xylose, L-arabinose, and sugar mixtures in lignocellulosic hydrolysates. This was achieved due to the developed novel method of positive selection of the mutants producing large colonies on plates with L-arabinose as the sole carbon and energy source combined with subsequent selection for resistance to 2-DG and BrPA. The best selected mutant, A107, accumulated over 20,91 g of ethanol/L in xylose-containing medium, which exceeds the wild-type strain’s ethanol accumulation by 50 times at the elevated temperature 45°C. However, it should be noted that previous studies reported an ethanol yield of 20.27 g/L at 45 °C for *K. marxianus.* However, the mentioned strain accumulated considerable levels of by-products, specifically xylitol and xylulose^18^.

**Fig. 6:**
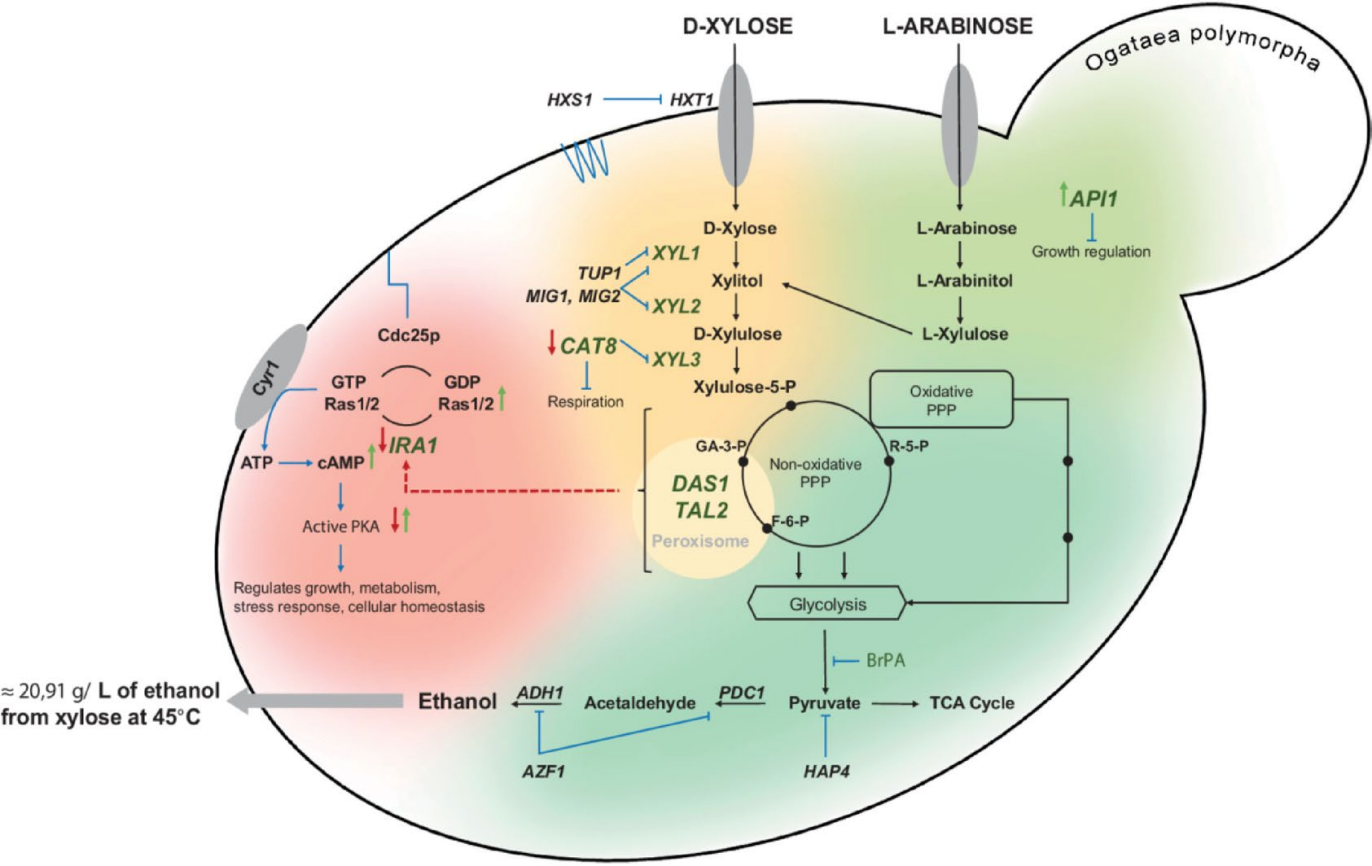
Genes involved in the regulation of pentose sugars alcoholic fermentation in *O. polymorpha* yeast. The schematic illustrates the metabolic pathways and key regulatory factors involved in alcoholic fermentation of pentose sugars in *O. polymorpha*. Enzymes and transporters include *XYL1* (xylose reductase), *XYL2* (xylitol dehydrogenase), *XYL3* (xylulokinase), *ADH1* (alcohol dehydrogenase), *PDC1* (pyruvate decarboxylase), *HXT1* (low-affinity hexose transporter), and *HXS1* (hexose-sensing receptor). Transcriptional regulators include *CAT8* and *AZF1* (transcription activators), *MIG1, MIG2, TUP1,* and *HAP4* (transcription factors). *DAS1* (dihydroxyacetone synthase) and *TAL2* (peroxisomal transketolase and transaldolase, respectively) play roles in pentose metabolism. The diagram also represents signal transduction pathways related to *IRA1* deletion and overexpression. Ras1/2 proteins mediate intracellular glucose signaling, while Cyr1 (adenylate cyclase) converts ATP to cAMP, activating PKA (protein kinase A), which regulates growth, metabolism, and stress responses. *IRA1* acts as a negative regulator of Ras1/2, facilitating the transition from the active (GTP-bound) to inactive (GDP-bound) state. The *API1* gene was identified in *O. polymorpha* as a gene involved in growth on L-arabinose, though its precise functional role remains to be fully elucidated. Arrows indicate regulatory interactions: red signifies inhibition/downregulation, green represents activation/upregulation, and blue highlights metabolic processes.

The development of our advanced strain was not limited to the selection for growth on L-arabinose but also involved targeted genetic modifications to optimize pentose metabolism. To prevent xylitol accumulation, a common byproduct in pentose fermentation, we overexpressed genes encoding key enzymes of the xylose catabolic pathway. Additionally, we inactivated *CAT8*, a transcriptional activator, to modulate metabolic flux. To further enhance sugar metabolism, we overexpressed *DAS1* and *TAL2*, which encode peroxisomal transketolase and transaldolase, respectively. Furthermore, engineering of xylose reductase reduced its affinity for NADPH, thereby improving cofactor balance^11–13^. In addition to these targeted modifications, the strain acquired non-identified mutations that conferred resistance to BrPA and impaired ethanol utilization^13^ (Fig. 6). Here, we paid attention to the unexplained yet substantial difference in ethanol production from two pentoses, xylose and L-arabinose, despite both supporting robust growth as sole carbon sources. One may assume that L-arabinose was partially converted to some intermediates of the catabolic pathway, e.g. arabitol, or alternatively converted primarily to biomass. This aspect of L-arabinose metabolism in the engineered strains of *O. polymorpha* is planned to be further studied.

Through mutant whole genome sequencing and reverse genetic approaches, we identified and provided the first direct evidence of novel genetic interactions between mutations in the *API1* and *IRA1* genes in *O. polymorpha* and the fermentation of xylose and L-arabinose (Fig. 6). Notably, the overexpression of *API1* led to a three-fold increase in biomass accumulation on L-arabinose as the sole carbon source and on the fermentation of this sugar; however, such modification had a less pronounced effect on biomass accumulation from xylose or its fermentation. The results suggest a novel function for the *API1* gene in *O. polymorpha*. The question remains on the possible reasons of activation of L-arabinose growth and fermentation by the mutated *API1* gene identified by us in A107 strain. We found that the observed mutation did not affect the expression level of *API1* (Supplementary Fig. 3) though phenotypically both mutated and overexpressed *API1* genes resulted to similar activation of L-arabinose fermentation (compare Figs. 2 and 3). We hypothesize that the mutated gene encodes catalytically more active D-arabinose-5-phosphae isomerase involved in some way in regulation of L-arabinose fermentation. This gene was not described before in yeasts and our data show that it not essential one. Although arabinose-5-phosphate isomerase is typically associated with bacterial metabolism, the gene identified in yeast might have evolved, with mutations potentially enabling the protein to play an unknown yet role in L-arabinose metabolism. Overexpression of the *API1* gene can e.g. lead to activation of L-arabinose entry into central metabolic pathways. Anyway, its specific involvement in L-arabinose (but not xylose) fermentation could be used for the construction of advanced L-arabinose-fermenting yeasts.

In contrast, the deletion of *IRA1* significantly increased biomass accumulation on xylose-containing medium (*P* = 0.002, one-way ANOVA, n = 3) and ethanol production during fermentation of this substrate (*P* = 0.05). In our previous work, we hypothesized that although *O. polymorpha* naturally ferments xylose, this occurs despite conflicting cellular signaling pathways^17^. Here, we demonstrated that deletion of the potential RAS/PKA inhibitor *IRA1* and subsequent reorganization of cellular signaling is crucial for improving growth and anaerobic fermentation of both xylose and L-arabinose. Snowdon et al. (2012) investigated the regulatory role of genes within the Ras/cAMP/PKA pathway in the degradation of the low-affinity glucose transporters Hxt3 and Hxt7 in *S. cerevisiae*. Their findings demonstrate that the activation of PKA inhibits the turnover and elimination of Hxt3 and Hxt7 from the plasma membrane under glucose-depleted conditions. In contrast, inactivation of the Ras/cAMP/PKA pathway is required to facilitate the turnover of these transporters, underscoring the pathway’s crucial role in modulating transporter stability in response to glucose availability^31,32^. Additionally, mutations in PKA regulators, particularly missense mutations and single base pair deletions or insertions, are high-frequency genetic events in the *IRA1* and *IRA2* loci and are frequently detected in laboratory evolution studies of strains with improved growth under various conditions^33,34^. The improved xylose consumption rate phenotype was reproduced by deletion of *IRA2*, confirming the role of cAMP/PKA signaling on xylose utilization in *S. cerevisiae*^35–37^.

The A107 mutant strain demonstrates the highest ethanol production from xylose, even compared to other our strains with genetic modifications such as BEP/*cat8∆/api1∆*, BEP/cat8Δ/API1*, BEP/*cat8∆/ira1∆*, BEP/cat8Δ/IRA1*. Nonsense mutation (as in strain A107) or knock out in the *IRA1* gene activate xylose and L-arabinose utilization and fermentation, however, worsen these parameters for glucose, the main sugar of lignocellulosic hydrolysate. Similar phenotypes have been reported in other studies, where the deletion of genes such as *IRA2*, *PDE1/2*, or *BCY1* of *S. cerevisiae*, which encodes negative regulators of PKA, enhances the xylose utilization rate, promotes biomass accumulation, and is associated with high ethanol yield from xylose. However, disruption of the cAMP-PKA pathway due to the loss of these genes also led to growth arrest under anaerobic conditions and poor aerobic growth on glucose. Complete deregulation of negative regulatory pathways cannot be considered viable, as such alterations result in reduced stress tolerance and lower biomass yields^38,39^.

Also, we confirmed that the Ras-cAMP-PKA pathway, specifically the *IRA1* gene in *O. polymorpha*, plays a crucial role in alleviating growth inhibition under stressful conditions such as high temperatures and low pH. Deletion of *IRA1* reduced stress tolerance, particularly at low pH, while its overexpression enhanced growth under extreme conditions, especially for xylose and L-arabinose utilization. During further strain development, it is important to reach a balanced improvement of the fermentation of at least both glucose and xylose. This is planned to be achieved using adaptive laboratory evolution as well as with positive selection with several potent inhibitors like glyoxylic acid, glucosamine, etc.^46^.

The A107 strain is a promising candidate for future SSF (simultaneous saccharification and fermentation) trials, as it demonstrates a technological advantage with enhanced ethanol productivity at elevated temperatures, in contrast to the wild-type *O. polymorpha* strains^3^ (Table 2). Furthermore, the A107 strain showed promising results in xylose fermentation, suggesting its applicability in lignocellulosic biomass conversion for bioethanol production. Still, L-arabinose fermentation should be substantially improved (at least 30–40 times) to be applicable commercially. Besides, tolerance to lignocellulose hydrolysate inhibitors needs to be elevated. Our forthcoming work will evaluate A107 in SSF, affirming its industrial potential for economically viable high-yield ethanol production from complex biomass sources and contributing to sustainable biofuel technology.

## Methods

### Strains and growth conditions

*Ogataea polymorpha* BEP/*cat8Δ*, BEP/*cat8Δ/DAS1/TAL2* and BEP/*cat8Δ/DAS1/TAL2*/107/2-DG/BrPA (A107) strains^12,13^ was grown on YPD (10 g/L yeast extract, 10 g/L peptone, and 20 g/L glucose or xylose, L-arabinose, or 50% bagasse hydrolysate) or minimal medium (1.7 g/L YNB without amino acids, 5 g/L ammonium sulfate or sodium nitrate, and 20 g/L glucose or other carbon sources) at 37 °C or 45 °C. Glucose, xylose, L-arabinose, ammonium sulfate, sodium nitrate, yeast extract, and peptone were purchased from Sigma-Aldrich (St. Louis, MO, USA). Sugarcane straw hydrolysate was prepared using liquid hot water pretreatment followed by enzymatic hydrolysis^40^ and provided in collaboration with Swedish Agricultural University, Uppsala, and GranBio AVAPCO, LLC (Thomaston, USA). The bagasse hydrolysate, with a pH of 5, contained glucose (48 g/L), xylose (23 g/L), galactose (0.1 g/L), L-arabinose (0.2 g/L), acetic acid (3 g/L), lactic acid (1.4 g/L) formic acid (0.93 g/L), furfural (0.62 g/L), and hydroxymethylfurfural (HMF) (0.43 g/L). Ethanol fermentation of *O. polymorpha* strains were tested as described previously^12^.

The *Escherichia coli* DH5α strain [*Φ80dlacZΔM15, recA1, endA1, gyrA96, thi-1, hsdR17(r − K, m* + *K), supE44, relA1, deoR, Δ(lacZYA–argF)U169*] was used as a host for plasmid propagation. Strain DH5α was grown at 37 °C in LB medium as described previously^41^. Transformed *E. coli* cells were maintained on a medium containing 100 mg/L of ampicillin.

## Induced mutagenesis methods

UV mutagenesis of *O. polymorpha* strains was carried out as previously described^42^, with non-irradiated samples used as controls to assess the survival rate of irradiated cells. After irradiation, cells were transferred to minimal media supplemented with 15% L-arabinose or D-arabinose; 15% L-arabinose and 200 mg/L 2-deoxy-D-glucose (2-DG); 15% L-arabinose, 200 mg/L 2-DG, and 0.05–0.11 mM 3-bromopyruvate (BrPA). 2-DG and BrPA were purchased from Merck (Darmstadt, Germany). 2-DG inhibits glycolysis by accumulating in cells as non-metabolizable phosphates^43^, potentially enhancing ethanol production through mutations in glycolysis-associated genes. BrPA is a synthetic analog of pyruvate with anti-tumor potential, known to inhibit ATP synthesis by targeting hexokinase, glyceraldehyde-3-phosphate dehydrogenase, and 3-phosphoglycerate kinase^44,45^. Apparently due to its inhibitory effects on multiple glycolytic enzymes, BrPA impairs growth on glucose or xylose substrates^13,46^.

Plates were incubated at 45 °C until colonies formed (approximately 2–4 days).

### DNA sequencing

Yeast cultures of BEP/*cat8Δ*, BEP/*cat8Δ/DAS1/TAL2*, and BEP/*cat8Δ/DAS1/TAL2/*107/2-DG/BrPA (A107) strains were harvested in the log phase from liquid YPD medium. Genomic DNA was extracted by the NucleoSpin Microbial DNA Mini kit (Macherey–Nagel, Germany) and sent to Explogen LLC (Ukraine) for sequencing library preparation. Sequencing libraries were prepared and indexed using the NovaSeq 6000 SP Reagent Kit v1.5 according to standard procedure (Illumina, USA). Paired-end libraries (2 × 150 bp) were sequenced on an Illumina NovaSeq 6000 (Novogene UK, United Kingdom).

An average of 3 million reads per genome was generated, providing approximately 100-fold genome coverage. Quality checks were done with FastQC v0.11.2^47^. The sequencing data were mapped to the reference genome using bwa 0.7.17^48^. The reads depth coverage analysis was performed using Qualimap (10.1093/bioinformatics/btv566). Reads mapping results were used to identify single-nucleotide polymorphisms (SNPs) and insertions and deletions (InDels) with Snippy 4.6.0^49^. De novo assembly of Illumina data was done using Newbler v3.0^50^.

### Construction and analysis of *api1∆* and *ira1∆ O. polymorpha* deletion mutants

For deletion, the 5′ and 3′ flanking regions of the *API1* and *IRA1* genes were amplified from the genomic DNA of wild-type *O. polymorpha* NCYC495 leu1-1 using primers RV_pl_015/RV_pl_016 and RV_pl_017/RV_pl_018 for *API1*, which are homologous to sequences up- and downstream of the 5’- and 3’-untranslated regions, and RV005/RV006 and RV007/RV008 for *IRA1*, respectively. All primers were synthesized by Genomed (Warsaw, Poland). The fragments were joined via overlap-PCR with primers RV_pl_015/RV_pl_018 and RV005/RV008. The 1925 bp and 1961 bp fragments were digested with SalI/NdeI and SacI/SalI, respectively, and cloned into a linearized pUC19 vector, leaving unique NotI and XbaI restriction sites between them. The *natNT2* gene, conferring nourseothricin resistance, was amplified from pUC19_GAPpr_GAPterm_NTC using primers RV_pl_013/RV_pl_014 and RV009/RV010, digested with NotI and XbaI, and cloned into the linearized plasmids. The constructed vectors were named pUC19_*api1∆* and pUC19_*ira∆* (Supplementary Table 5). The correctness of the plasmids was verified by restriction analysis. All restriction endonucleases, DNA polymerase, DNA ligase, and Gibson assembly utilized in molecular biology experiments were obtained from New England Biolabs (NEB, Ipswich, MA, USA) or Thermo Fisher Scientific (Waltham, MA, USA). The constructed plasmids were introduced into the *O. polymorpha* BEP/*cat8∆* strain by electroporation using a Bio-Rad Gene Pulser (Bio-Rad, Hercules, CA, USA)^51^. Transformants were selected on YPD medium supplemented with nourseothricin (100 mg/L). After two days of incubation, stable transformants were isolated by culturing under non-selective conditions. The deletion of the respective genes was verified through PCR performed on an Eppendorf® Mastercycler® Nexus X2 Thermal Cycler (Eppendorf, Hamburg, Germany). Primers homologous to sequences up- and downstream of the 5′- and 3′-untranslated regions of the *API1* and *IRA1* genes, as well as primers specific to the selective marker, were used: RV_pl_019/RV012 and RV020/RV_pl_020 for *API1*, and RV018/RV012 and RV019/RV020 for *IRA1*. The primer pairs used for PCR are listed in Supplementary Table 3. The DNA fragments of the expected sizes, generated by PCR, confirmed the deletion of the target genes. Constructed strains were designated as BEP/*cat8Δ/api1Δ* and BEP/*cat8Δ/ira1Δ*.

### Construction and analysis of *O. polymorpha* strains with overexpression of *API1* and *IRA1* genes

The *API1* gene was amplified from the genomic DNA of the wild-type strain *O. polymorpha* NCYC495 leu1-1 using PCR with the primer pair RV001/RV002. The resulting 1119 bp fragment was digested with XbaI/NotI restriction endonucleases and cloned into a pre-constructed plasmid, pUC19_GAPpr_GAPterm_NTC^17^. This plasmid contains a strong constitutive promoter of the *GAP1* gene from *O. polymorpha*, encoding glyceraldehyde-3-phosphate dehydrogenase, and a *natNT2* marker gene conferring resistance to nourseothricin. Similarly, for the overexpression of the *IRA1* gene, fragment A (2098 bp) of *IRA1_Op* was amplified from total DNA using PCR with primers RV_pl_aF_30/aR_31. Fragment B (2058 bp) of *IRA1_Op* was amplified using primers RV_pl_bF_32/RV_pl_bR_33. Fragments C and D, 2026 bp and 2195 bp in size, were amplified with RV_pl_cF_34/cR_35 and RV_pl_dF_36/dR_37, respectively. To avoid restriction site scars, all fragments and the pUC19_GAPpr_GAPterm_NTC vector (sequentially digested with XbaI/NotI) were assembled using Gibson DNA assembly^52^. The accuracy of the constructed vectors pUC19_GAPpr_*API1*_GAPterm_NTC and pUC19_GAPpr_*IRA1*_GAPterm_NTC was verified by restriction and PCR analysis using primers RV_pl_38/39 and RV_pl_40/41. Additionally, a conditional recombinant strain with overexpression of *IRA1* was constructed by replacing the endogenous promoter of the *IRA1* gene with the regulated promoter of the *YNR1* gene, which encodes nitrate reductase and is repressed by ammonium sulfate when used as a nitrogen source. The YNR1 promoter was amplified from the genomic DNA of the *O. polymorpha* NCYC495 leu1-1 strain using primers RV_pl_57/RV_pl_66. The native terminator of the *IRA1* gene was amplified using primers RV_pl_67/RV_pl_68. The obtained DNA fragments were assembled by PCR overlap using primers RV_pl_57 and RV_pl_68. The resulting fragment (1.2 kb) was digested with SacI/SalI and cloned into the corresponding sites of the pUC19 vector. The selective marker gene providing resistance to nourseothricin was amplified with primers RV_pl_61 and RV_pl_62 and cloned into the NdeI/EcoRI sites of the plasmid. After digestion with the restriction endonucleases NheI/NotI, the *IRA1* gene amplified with primers RV_IRA_F/RV_IRA_R was cloned into the vector. The correctness of the constructed vector was verified by restriction and PCR analysis using primers RV_YNR_ira_check_F/RV_YNR_ira_check_R. The constructed plasmids were introduced into BEP/cat8∆ strains via electroporation^51^. Transformants were selected on YPD medium supplemented with nourseothricin (100 mg/L). On the second day of cultivation, a series of transformants were selected. To obtain stable recombinant strains, transformants were cultured under non-selective conditions, followed by selection of clones that retained the ability to grow on antibiotic-containing medium. In stable transformants, plasmid integration into the genome was confirmed by PCR using primers RV003/RV004 for BEP/cat8∆/API1 strains. Recombinant BEP/cat8∆/*IRA1* strains containing the vector pUC19_GAPpr_*IRA1*_GAPterm_NTC or pUC19_YNRpr_*IRA1*_IRA1term_NTC were not successfully obtained (Supplementary Fig. 5). To reduce the plasmid size and increase the transformation frequency, the likelihood of successful transformation, particularly in yeast, where larger plasmids have lower efficiency, unnecessary elements were removed from the plasmids. The amplified GAPpr_*IRA1*_GAPterm_NTC (10.6 kb) and YNRpr_*IRA1*_IRA1term_NTC (10.5 kb) fragments using primers RV_pl_73/RV_pl_74 and RV_pl_71/RV_pl_72, were transformed into BEP/cat8∆ strains via electroporation. In the resulting stable transformants, integration of the fragment into the genome was confirmed by PCR using primer pairs RV_pl_38/ RV_pl_39 or RV_pl_40/ RV_pl_41, respectively.

In this study, an asterisk (*) next to a gene symbol denotes its overexpression. For example, API1* represents the overexpressed allele, while *API1* without an asterisk denotes the wild-type allele. This notation also applies to the *IRA1* gene.

### Real-time quantitative PCR (qRT-PCR)

The copy numbers and elative expression levels of *API1* and *IRA1* genes were determined using real-time qPCR (Table 1). The qRT-PCR was performed by the 7500 Fast Real-Time PCR System (Applied Biosystems, Foster City, CA, USA) with the SG OneStep qRT-PCR kit (EURx Ltd., Gdansk, Poland) using gene-specific pairs of primers, RNA as a template, and ROX reference passive dye according to the manufacturer’s instructions as described previously^12^. The primer pairs are listed in Supplementary Table 3. *ACT1* was used as the reference gene. The ΔΔCt values were normalized to the control sample, which was assigned a value of 1.0. Statistical analysis is described in the “Statistical analysis” Section.

### Analytical methods

The biomass was determined turbidimetrically using a Helios Gamma spectrophotometer (Thermo Fisher Scientific, Waltham, MA, USA) at an optical density (OD) of 590 nm in a 10 mm cuvette, with gravimetric calibration. Concentrations of xylose, L-arabinose, glucose, various components of hydrolysates, and ethanol from fermentation in the medium were analyzed by HPLC PerkinElmer Series 200 (Waltham, MA, USA) using an Aminex HPX-87H ion-exchange column (Bio-Rad, Hercules, CA, USA). A mobile phase of 4 mM H₂SO₄ was used at a flow rate of 0.6 mL/min, and the column temperature was set to 35 °C. Alternatively, ethanol concentrations in the medium were determined using an alcohol oxidase/peroxidase-based enzymatic kit “Alcotest”^53^. Experiments were performed at least three times.

The sugar consumption rate (SQR) for each strain was calculated using the following formula: SQR = ΔCs/(Δt × OD), where, ΔCs is the change in sugar concentration (g/L) over the time interval Δt; Δt is the time (hours) during which sugar consumption was measured; OD is the optical density of the culture at the time of measurement, used to normalize for cell density. Sugar concentrations were determined using HPLC method and optical density was measured with a spectrophotometer at 590 nm. The sugar consumption rate was calculated for each time point, and average values were computed for each fermentation stage for each strain^54^.

The specific growth rate (μ) was calculated during the exponential phase of growth (between 24 and 48 h) using the natural logarithm of optical density (OD_590_) values, according to the formula: μ = (ln OD_2_ – ln OD_1_)/(t_2_ − t_1_), where OD_1_ and OD_2_ represent optical density values at time points t_1_ and t_2_ (in hours), respectively^55^.

### Statistical analysis

For each dataset, the mean, standard deviation (SD), and standard error of the mean (SE) were calculated based on three independent biological replicates (n = 3). The 95% confidence intervals (CI) were derived from the SE values using the *t-*distribution, with a critical *t*-value of 4.303 (degrees of freedom = 2, α = 0.05). All values in the tables are reported as mean ± 95% CI, unless otherwise indicated. Statistical significance was assessed using one-way analysis of variance (one-way ANOVA) for comparisons among multiple groups. Due to the limited sample size (n = 3), normality was assumed. Additionally, unpaired two-tailed *t*-tests with unequal variance were used for pairwise comparisons. The analyses were performed using Microsoft Excel 2016 (Microsoft Corp., Redmond, WA, USA) with the Analysis ToolPak add-in, and PAST software^56^. *P* values < 0.05 were considered statistically significant. Exact *P* values are reported in the main text. For graphical representation, error bars represent standard error of the mean (SE), and statistical significance is indicated by asterisks: * *P* ≤ 0.05, ** *P* ≤ 0.01, *** *P* ≤ 0.001 (Fig. 5)^57^.

## Electronic supplementary material

Below is the link to the electronic supplementary material.

Supplementary Material 1

## Data Availability

All datasets supporting the conclusions of this study are available within the article and its supplementary files. The sequencing data generated in this study have been deposited in the European Nucleotide Archive (ENA) at the European Bioinformatics Institute (EBI) under project number PRJEB85262, including whole-genome sequencing of *Ogataea polymorpha* yeast strains BEP/cat8Δ and its derivatives [https://www.ebi.ac.uk/ena/browser/view/PRJEB85262]. Additional datasets, including raw fermentation and growth data, have been deposited on Zenodo [10.5281/zenodo.15571557].
