# Supplementary material for: Positive selection of efficient ethanol producers from xylose at 45 °C in the yeast Ogataea polymorpha
Source: Sci Rep. 2025 Jul 22;15:26530. doi: 10.1038/s41598-025-12204-2 (PMC12280177; doi:10.1038/s41598-025-12204-2)
Supplement: Supplementary file 1 — Supplementary Material 1 [file 41598_2025_12204_MOESM1_ESM.pdf]

Positive selection of efficient ethanol producers from xylose at 45°C in the yeast *Ogataea polymorpha*

Roksolana Vasylyshyn, Justyna Ruchala, Kostyantyn Dmytruk and Andriy Sibirny

Supplementary Fig. 1 Sugar consumption dynamics during high-temperature alcoholic fermentation at 45 °C.

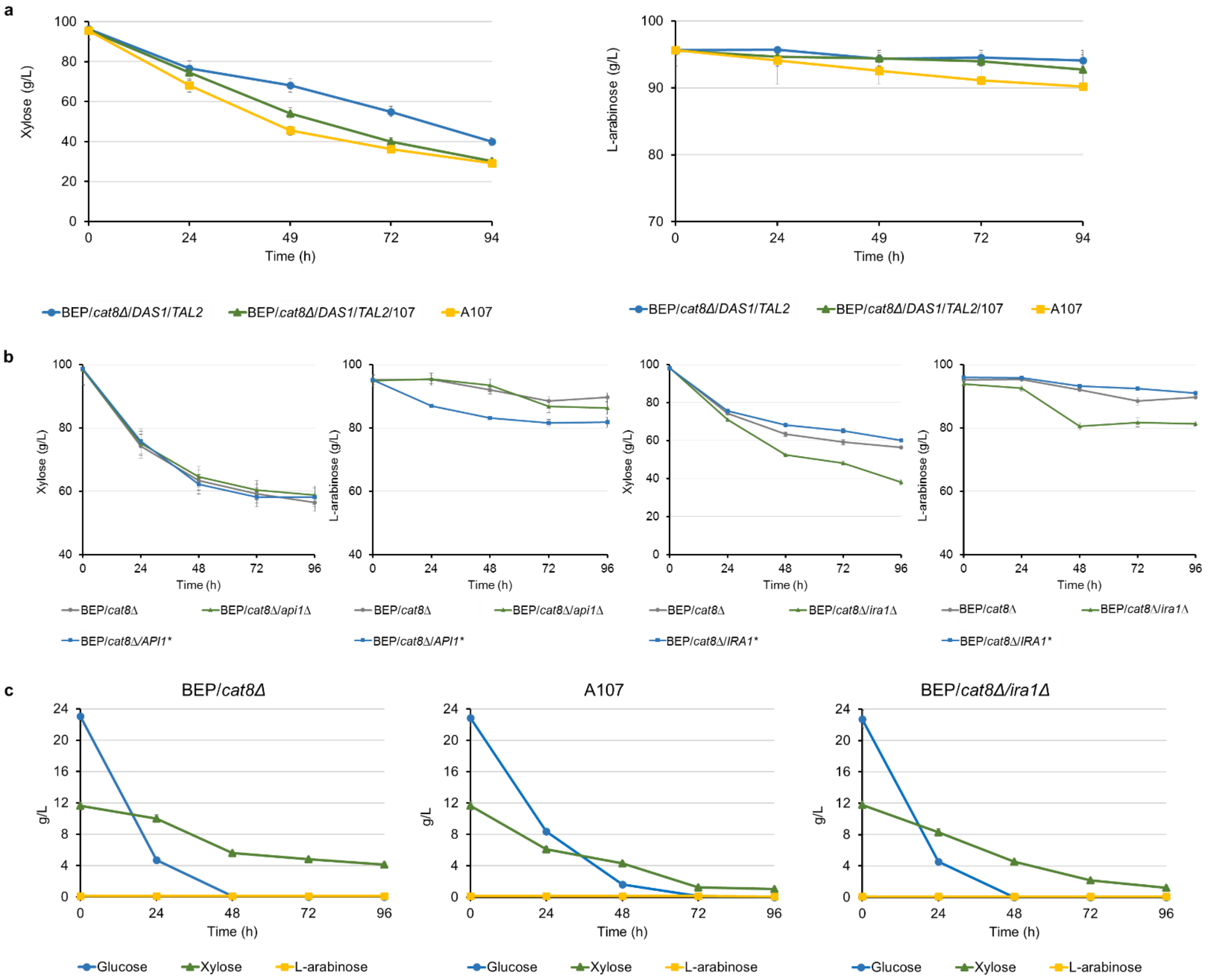

(**a**) Sugar consumption dynamics by the parental BEP/*cat8Δ/DAS1/TAL2* strain and selected mutants BEP/*cat8Δ/DAS1/TAL2*/107/2-DG and BEP/*cat8Δ/DAS1/TAL2*/107/2-DG/BrPA (A107) during high-temperature alcoholic fermentation of 10% xylose or 10% L-arabinose. (**b**) Sugar consumption dynamics during high-temperature alcoholic fermentation at 45 °C in the medium with 10% xylose, 10% L-arabinose by the parental BEP/*cat8Δ* strain and obtained BEP/*cat8Δ/api1Δ*, BEP/*cat8Δ/API1**, BEP/*cat8Δ/ira1Δ*, BEP/*cat8Δ/IRA1** strains or (**c**) 50% bagasse hydrolysate by the parental BEP/cat8 strain and obtained A107, BEP/cat8Δ/ira1Δ strains. Error bars represent the standard error of the mean (SE), n = 3. Details of SE calculation are provided in the "Methods" section.

Supplementary Table 1. List of identified changes that occurred in the genome of the obtained yeast strain A107.

TYPE	EFFECT	GENE	PRODUCT
snp	missense_variant c.423410G>C p.Leu2Phe	araA	Uncharacterized protein in yeast
ins	frameshift_variant c.958485_958486insGT	YBR140C	IRA1 (YBR140C) and IRA2 (YOL081W)
snp	stop_lost c.950G>C p.Ter317Serext*?	-	Uncharacterized protein HP_1429(HP_1429)
snp	intron_variant c.389-34C>T	HTA2	Histone H2A.1(HTA2)
snp	missense_variant c.827G>T p.Ser276Ile		Fungal specific transcription factor domain
complex	intergenic_region n.2415699_2415700delGAinsAC	-	-

Supplementary Fig. 2.

(**a**) Alignment of the API1 protein sequence in the parental *O. polymorpha* BEP/*cat8Δ/DAS1/TAL2* strain and mutant A107. (**b**) Fragment alignment of the IRA1 protein sequence in the parental *O. polymorpha* BEP/*cat8Δ/DAS1/TAL2* strain and mutant A107.

a

```
Sequence 1 length:372
Sequence 2 length:372
Alignment length: 372
Identity:     371/372 (99.73%)
Similarity:   371/372 (99.73%)
Gaps:           0/372 (0.00%)

1     M I R R G S E L S F H I E D D T S P L L E D A V R R V D S I L A S Q N R A I S Y L V G Q Y R N S K W S Q D H M K A A L K     60
      | | | | | | | | | | | | | | | | | | | | | | | | | | | | | | | | | | | | | | | | | | | | | | | | | | | | | | | | | | | |
1     M I R R G S E L S F H I E D D T S P L L E D A V R R V D S I L A S Q N R A I S Y L V G Q Y R N S K W S Q D H M K A A L K     60

61    I L N N S L N D G G K V I V S G I G K S Y K I A G K T V A T L N S L S V H S A L L H P S E A L H G D L G I I R E D H H D    120
      | | | | | | | | | | | | | | | | | | | | | | | | | | | | | | | | | | | | | | | | | | | | | | | | | | | | | | | | | | | |
61    I L N N S L N D G G K V I V S G I G K S Y K I A G K T V A T L N S L S V H S A L L H P S E A L H G D L G I I R E D H H D    120

121   S L L I I S A S G N S P E L T T L L E Y V P A S V P V V L V T C T K V S A L S K H P K V N A L F Y A E L P P K V S E K N    180
      | | | | | | | | | | | | | | | | | | | | | | | | | | | | | | | | | | | | | | | | | | | | | | | | | | | | | | | | | | | |
121   S L L I I S A S G N S P E L T T L L E Y V P A S V P V V L V T C T K V S A L S K H P K V N A L F Y A E L P P K V S E K N    180

181   L Y G L Q A P T I S T T I C L T L L D G I S I A L S E L H I R D L E V R Q K R F G D R H P G G A I G L H Y L Q D L D Q T    240
      | | | | | | | | | | | | | | | | | | | | | | | | | | | | | | | | | | | | | | | | | | | | | | | | | | | | | | | | | | | |
181   L Y G L Q A P T I S T T I C L T L L D G I S I A L S E L H I R D L E V R Q K R F G D R H P G G A I G L H Y L Q D L D Q T    240

241   R R L S G S N G S T S E C S S T E K S V S E A S S E S V Y E Q P N L Q D D D F G D A N D I L E L P N L K L L N E I K L S    300
      | | | | | | | | | | | | | | | | | | | | | | | | | | | | | | | | | | | | | | | | | | | | | | | | | | | | | | | | | | | |
241   R R L S G S N G S T S E C S S T E K S V S E A S S E S V Y E Q P N L Q D D D F G D A N D I L E L P N L K L L N E I K L S    300

301   V S K I T L P K V P G D E L E L L R L A L M Y D F L L V M N A E T N R G I R T K N L L D I Y R K G K L Q E D S W D E I L    360
      | | | | | | | | | | | | | | | |   | | | | | | | | | | | | | | | | | | | | | | | | | | | | | | | | | | | | | | | | | |
301   V S K I T L P K V P G D E L E L F R L A L M Y D F L L V M N A E T N R G I R T K N L L D I Y R K G K L Q E D S W D E I L    360

361   W K I N D S F I K L S L                                                                                                  372
      | | | | | | | | | | | |
361   W K I N D S F I K L S L                                                                                                  372
```

b

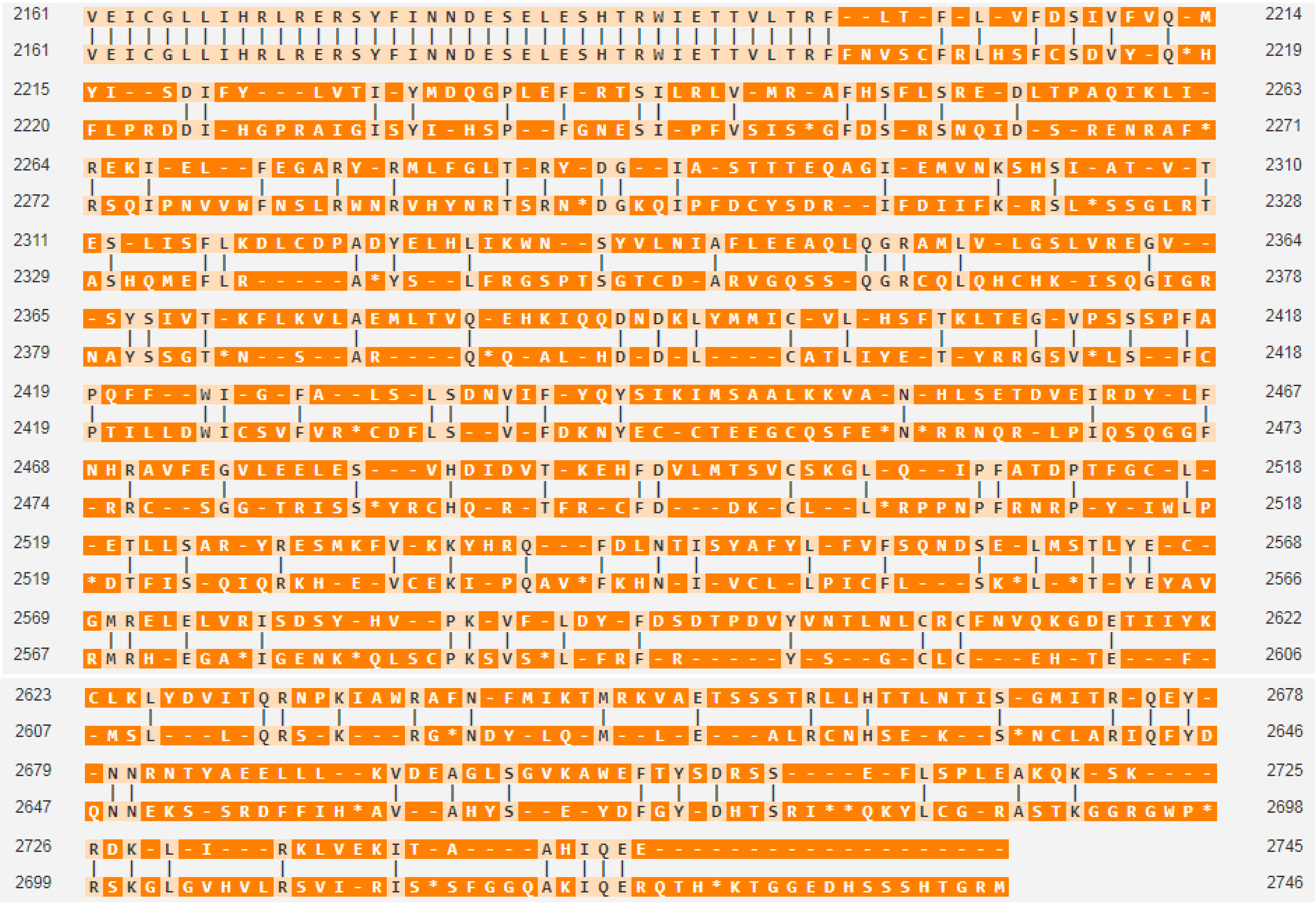

2161 VEICGLLIHRLRERSYFINNDESELESHTRWIETTVLTRF--LT-F-L-VFDSIVFVQ-M 2214
2161 VEICGLLIHRLRERSYFINNDESELESHTRWIETTVLTRFFNVSCFRLHSFCSDVY-Q*H 2219
2215 YI--SDIFY---LVTI-YMDQGPLEF-RTSILRLV-MR-AFHSFLSRE-DLTPAQIKLI- 2263
2220 FLPRDDI-HGPRAIGISYI-HSP--FGNESI-PFVSIS*GFDS-RSNQID-S-RENRAF* 2271
2264 REKI-EL--FEGARY-RMLFGLT-RY-DG--IA-STTTEQAGI-EMVNKSHSI-AT-V-T 2310
2272 RSQIPNVVWFNSLRWNRVHYNRTSRN*DGKQIPFDCYSDR--IFDIIFK-RSL*SSGLRT 2328
2311 ES-LISFLKDLCDPADYELHLIKWN--SYVLNIAFLEEAQLQGRAMLV-LGSLVREGV-- 2364
2329 ASHQMEFLR-----A*YS--LFRGSPTSGTCD-ARVGQSS-QGRCQLQHCHK-ISQGIGR 2378
2365 -SYSIVT-KFLKVLAEMLTVQ-EHKIQQDNDKLYMMIC-VL-HSFTKLTEG-VPSSSPFA 2418
2379 NAYSSGT*N--S--AR----Q*Q-AL-HD-D-L----CATLIYE-T-YRRGSV*LS--FC 2418
2419 PQFF--WI-G-FA--LS-LSDNVIF-YQYSIKIMSAALKKVA-N-HLSETDVEIRDY-LF 2467
2419 PTILLDWICSVFVR*CDFLS--V-FDKNYEC-CTEEGCQSFE*N*RRNQR-LPIQSQGGF 2473
2468 NHRAVFEGVLEELES---VHDIDVT-KEHFDVLMTSVCSKGL-Q--IPFATDPTFGC-L- 2518
2474 -RRC--SGG-TRISS*YRCHQ-R-TFR-CFD---DK-CL--L*RPPNPFRNRP-Y-IWLP 2518
2519 -ETLLSAR-YRESMKFV-KKYHRQ---FDLNTISYAFYL-FVFSQNDSE-LMSTLYE-C- 2568
2519 *DTFIS-QIQRKH-E-VCEKI-PQAV*FKHN-I-VCL-LPICFL---SK*L-*T-YEYAV 2566
2569 GMRELELVRISDSY-HV--PK-VF-LDY-FDSDTPDVYVNTLNLCRCFNVQKGDETIIYK 2622
2567 RMRH-EGA*IGENK*QLSCPKSVS*L-FRF-R-----Y-S--G-CLC---EH-TE---F- 2606
2623 CLKLYDVITQRNPKIAWRAFN-FMIKTMRKVAETSSSTRLLHTTLNTIS-GMITR-QEY- 2678
2607 -MSL---L-QRS-K---RG*NDY-LQ-M--L-E---ALRCNHSE-K--S*NCLARIQFYD 2646
2679 -NNRNTYAEELLL--KVDEAGLSGVKAWEFTYSDRSS----E-FLSPLEAKQK-SK---- 2725
2647 QNNEKS-SRDFFIH*AV--AHYS--E-YDFGY-DHTSRI**QKYLCG-RASTKGGRGWP* 2698
2726 RDK-L-I---RKLVEKIT-A----AHIQEE---------------------- 2745
2699 RSKGLGVHVLRSVI-RIS*SFGGQAKIQERQTH*KTGGEDHSSSHTGRM 2746

Supplementary Fig. 3.

Relative expression levels of *API1* and *IRA1* genes expression in A107 strain versus the recipient strain BEP/*cat8Δ/DAS1/TAL2* at the third day of (**a**) 10% xylose or (**b**) 10% L-arabinose fermentation at 45°C. Values represent mean ± SE from three biological replicates. Details of SE calculation are provided in the "Methods" section.

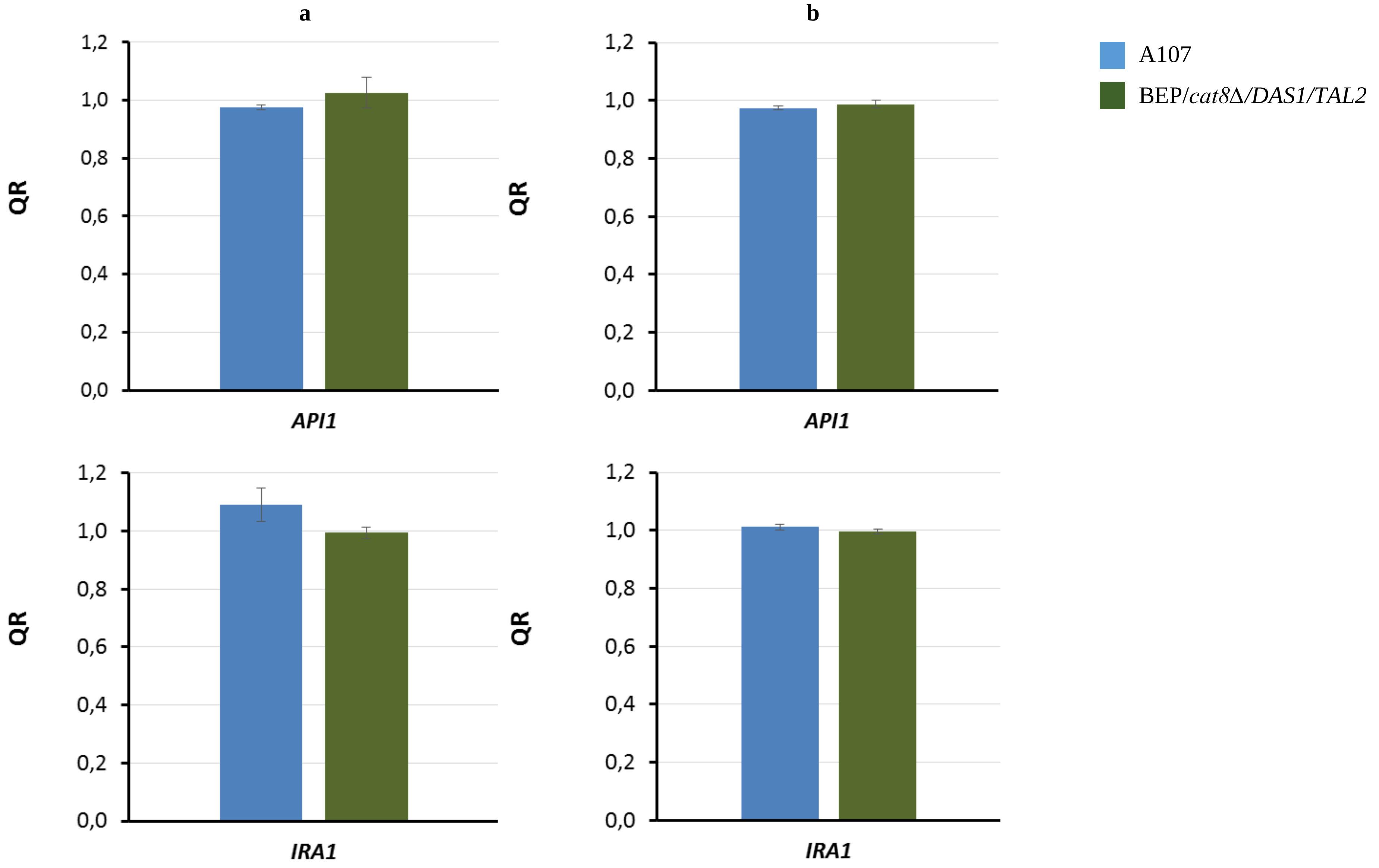

Supplementary Fig. 4. Growth and ethanol production of the strains overexpressing *IRA1* under the control of the nitrate reductase YNR1 promoter.

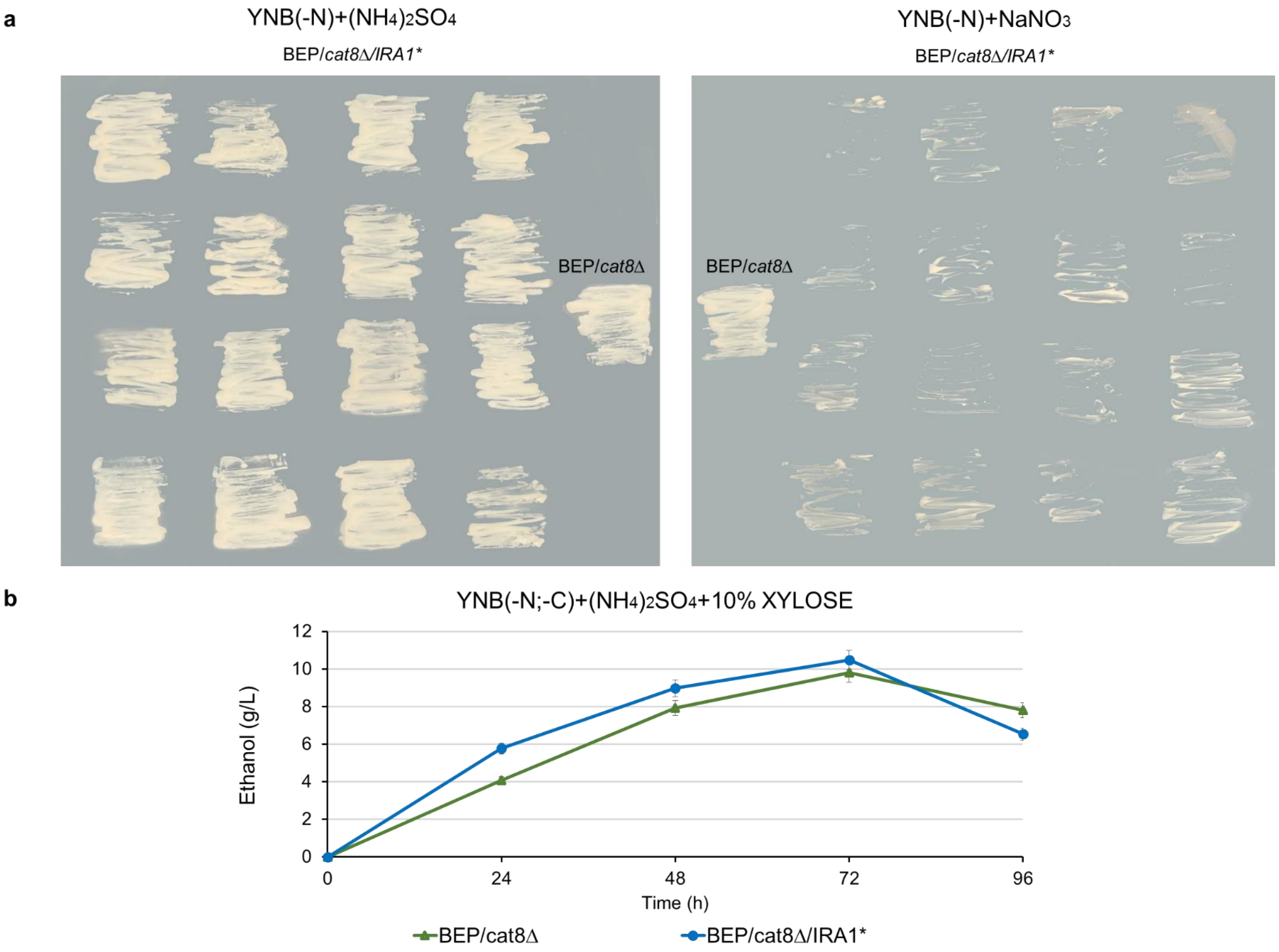

(**a**) Growth of the parental *O. polymorpha* strain BEP/cat8Δ and strains overexpressing *IRA1* under the control of the nitrate reductase YNR1 promoter in media with 2% xylose and either ammonium sulfate or nitrate. (**b**) Ethanol production during high-temperature alcoholic fermentation at 45°C from 10% xylose in ammonium sulfate media for BEP/*cat8Δ* and BEP/*cat8Δ/IRA1** strains under the control of YNR1 promoter. Error bars represent the standard error of the mean (SE), n = 3. Details of SE calculation are provided in the “Methods” section.

Supplementary Table 2. Specific growth rates (μ, h^{-1}) of *O. polymorpha* strains cultivated in media with varying xylose concentrations (2%–25%) over the 14-24 h; 24-48 h; 14–72 h time interval.

Strains included the parental strain BEP/*cat8*Δ, reference strain A107, and engineered strains BEP/*cat8Δ/API1** and BEP/*cat8Δ/ira1*Δ. Growth was monitored by measuring optical density at 590 nm (OD_{590}), and μ values were calculated based on the natural logarithm of biomass increase during the exponential phase (**a)** 14-24 h, (**b)** 24-28 h or as an overall growth rate across the full 14–72 h period. Average growth rate over 14–72 h, including non-exponential phases, shown for reference only. The calculation method is detailed in the "Methods" section.

a

Strain	2%	8%	10%	15%	20%	25%
**BEP/cat8Δ**	0.0955	0.0557	0.0619	0.0350	0.1004	0.0314
**A107**	0.0828	0.0632	0.0628	0.0328	0.0474	0.0389
**BEP/cat8Δ/*API1****	0.0680	0.0592	0.0734	0.0595	0.0853	0.0231
**BEP/cat8Δ/ira1Δ**	0.0557	0.0552	0.0443	0.0396	0.0488	0.0329

b

Strain	2%	8%	10%	15%	20%	25%
**BEP/cat8Δ**	0.0223	0.0340	0.0348	0.0675	0.0585	0.0565
**A107**	0.0537	0.0420	0.0558	0.0437	0.0443	0.0788
**BEP/cat8Δ/*API1****	0.0538	0.0376	0.0477	0.0695	0.0605	0.0527
**BEP/cat8Δ/ira1Δ**	0.0434	0.0354	0.0400	0.0364	0.0419	0.0657

c

Strain	2%	8%	10%	15%	20%	25%
**BEP/cat8Δ**	0.0304	0.0247	0.0264	0.0386	0.0571	0.0567
**A107**	0.0430	0.0297	0.0359	0.0296	0.0318	0.0466
**BEP/cat8Δ/*API1****	0.0404	0.0264	0.0352	0.0437	0.0530	0.0538
**BEP/cat8Δ/ira1Δ**	0.0350	0.0265	0.0274	0.0273	0.0266	0.0388

Supplementary Table 3. List of primers used in this study.

PRIMER NAME	PRIMER SEQUENCE 5'-3
RV001	ATTCTAGAATGATTAGGAGAGGTTCAGAGC
RV002	ATTCTAGAGCG GCCGCTTATAAACTTAACTTGATAAAAGAGTC
RV003	GTCATACGTGTAGGTTTTTGGC
RV004	GCTGGCACATATTCAAGTAGC
RV005	ATGAGCTCATGTCATCTATAGTACAGGAGGCCGATCAAACAG
RV006	ATGAACCCCTTCGGTAAGTTTCTCTAGAGAGAGAGAGTACGCTTTGAC
RV007	ATGTCAAAGCGTACTCTCTCTCTCTAGAGAAACTTACCGAAGGGGTTC
RV008	ATGTCGACTCATTCTTCCTGTATGTGAGCTGCTGTGATC
RV009	ATTCTAGACTTAACTATGCGGCATCAGAG
RV010	ATTCTAGACCGAGATTCATCAACTCATTGC
RV012	GAAAGAAGAACCTCAGTGGC
RV018	CCATCCCAAAGAGAGGAAGC
RV020	GCCACTGAGGTTCTTCTTTC
RV_pl_013	ATGCGGCCGCCTTAACTATGCGGCATCAGAG
RV_pl_014	ATGCGGCCGCCCGAGATTCATCAACTCATTGC
RV_pl_015	ATGTCGACAGAATCATTGGGGAAACTAGAACCAAGATATTACTCAATAGC
RV_pl_016	ATCAACAAGTTCTTCGTTCTGAGCGGCCGCATATCGCTCTGTTTTGACTG
RV_pl_017	ATCAGTCAAAACAGAGCGATATGCGGCCGCTCAGAACGAAGAACTTGTTG
RV_pl_018	ATCAT ATGTTCGCCTATCTTGTCAAAAAACTTTCCTATAAACCACTCG
RV_pl_019	CCGATGTCCCATTTTCTTTTCTTC
RV_pl_rt_24	TCAGAACGAAGAACTTGTTGG
RV_pl_rt_25	TTATAAACTTAACTTGATAAAAGAG
RV_pl_rt_26	CGAGACTTCTTCATCCACTAGGC
RV_pl_rt_27	TGAGTACGTGAACTCCCAGGC
RV_pl_aF_30	GTACAAAGATAATATAGAAACAAATCTAGAATGTCATCTATAGTACAGGAGGCCGATCAAAC
RV_pl_aR_31	CATTGCTATGGCTCGTGATAATGCTAGAAGTAGAC
RV_pl_bF_32	GTCTACTTCTAGCATTATCACGAGCCATAGCAATG
RV_pl_bR_33	TTGGCAAAATCGTCAACCTCTGAAGTAGGACAG
RV_pl_cF_34	CTGTCCTACTTCAGAGGTTGACGATTTTGCCAA
RV_pl_cR_35	ATTAGCGGCAAGCTGGTCAGATAGAGCAAAGATA
RV_pl_dF_36	TATCTTTGCTCTATCTGACCAGCTTGCCGCTAAT

RV_pl_dR_37	AGATGGAGCCGAGCCTCGAGCCCGGGGCGGCCGCTCATTCTTCCTGTATGTGAGCTGCTGTGATCTTC
RV_pl_38	AGTCATACGTGTAGGTTTTTGGCG
RV_pl_39	GTCAAATTGATTGATGTGTTCGATAGTG
RV_pl_40	TCAAAAAGGGGATGAAACGATTATTTAC
RV_pl_41	GCAAGCAACGATAGTGTTTTTGAAC
RV_pl_57	ATGTCGACTTCTTTCTTGCTACGAAATGGGC
RV_pl_61	ATGAATTCCTTAACTATGCGGCATCAGAGC
RV_pl_62	ATCATATGCAGGGCATGCTCATGTAGAGC
RV_pl_66	GAAAATCATTTTTTTCGTAGCATTATGCGGCCGCGCTAGCTACGACTATCCGGAATTCTTGAG
RV_pl_67	CTCAAGAATTCCGGATAGTCGTAGCTAGCGCGGCCGCATAATGCTACGAAAAAAATGATTTTC
RV_pl_68	AT GAGCTC ATTGTTTAGTTGACTACCCATTCG
RV_IRA_F	ATGCTAGCTCTAGAATGTCATCTATAGTACAGGAGGC
RV_IRA_R	ATGCGGCCGCTCATTCTTCCTGTATGTGAGC
RV_YNR_ira_check_F	TTTATTCTCCGTCTTATCTTGC
RV_YNR_ira_check_R	CTGAACAGCCTGTATACAGC
RV_pl_71	ATTTCTTTCTTGCTACGAAATGGGC
RV_pl_72	ATCAGGGCATGCTCATGTAGAGC
RV_pl_73	ATCTTAACTATGCGGCATCAGAGC
RV_pl_74	ATCTGCCACGAGGTACCACAAAG
IRA1X_F_rt	CAGAAATACTTATGCGGAAGAGC
IRA1X_R_rt	CATTCTTCCTGTATGTGAGCTGC

Supplementary Fig. 5. Vector maps used in this study

Vector maps used in this study: pUC19/GAPpr/*API1*_Op/NTC, pUC19_GAPpr_*IRA1*_GAPterm_NTC, pUC19_YNRpr_*IRA1*_IRA1term_NTC, pUC19/*apiΔ* and pUC19/*ira1Δ*.

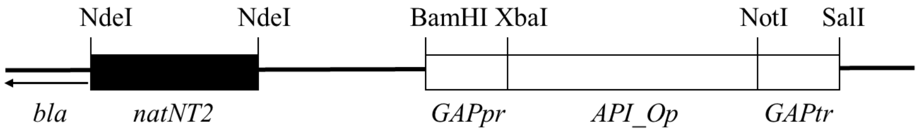

pUC19/GAPpr/*API1_Op*/NTC ~5,9kb

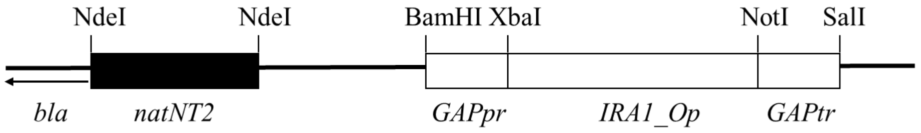

pUC19/GAPpr/*IRA1_Op*/NTC ~13kb

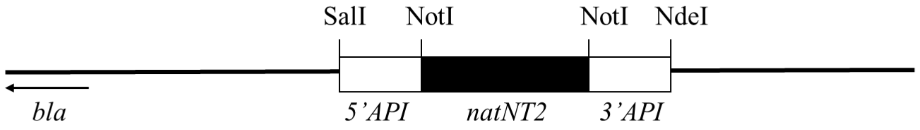

pUC19/*api1Δ* ~5,6kb

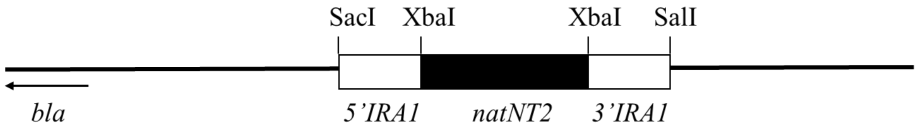

pUC19/*ira1Δ* ~5,6kb

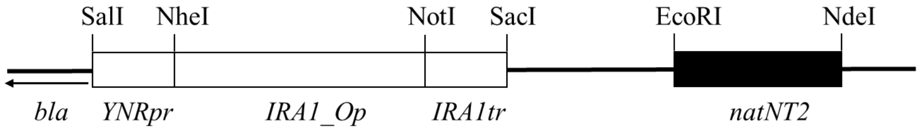

pUC19/YNRpr/*IRA1_Op*/NTC ~13kb
